# Pretrichodermamides D–F from a Marine Algicolous Fungus *Penicillium* sp. KMM 4672

**DOI:** 10.3390/md14070122

**Published:** 2016-06-27

**Authors:** Anton N. Yurchenko, Olga F. Smetanina, Elena V. Ivanets, Anatoly I. Kalinovsky, Yuliya V. Khudyakova, Natalya N. Kirichuk, Roman S. Popov, Carsten Bokemeyer, Gunhild von Amsberg, Ekaterina A. Chingizova, Shamil Sh. Afiyatullov, Sergey A. Dyshlovoy

**Affiliations:** 1 G.B. Elyakov Pacific Institute of Bioorganic Chemistry, Far Eastern Branch of the Russian Academy of Sciences, Prospect 100-letiya Vladivostoka, 159, Vladivostok 690022, Russia; smetof@rambler.ru (O.F.S.); kaaniv@piboc.dvo.ru (A.I.K.); 161070@rambler.ru (Y.V.K.); sheflera@bk.ru (N.N.K.); prs_90@mail.ru (R.S.P.); martyyas@mail.ru (E.A.C.); afiyat@piboc.dvo.ru (S.S.A.); dyshlovoy@gmail.com (S.A.D.); 2 School of Natural Science, Far Eastern Federal University, Sukhanova St., 8, Vladivostok 690000, Russia; ev.ivanets@yandex.ru; 3 Laboratory of Experimantal Oncology, Department of Oncology, Hematology and Bone Marrow Transplantation with Section Pneumology, Hubertus Wald-Tumorzentrum, University Medical Center Hamburg-Eppendorf, Hamburg 20246, Germany; cbokemeyer@uke.de (C.B.); g.von-amsberg@uke.de (G.A.)

**Keywords:** marine-derived fungus, secondary metabolites, diketopiperazine, oxazadecaline, cytotoxicity

## Abstract

Three new epidithiodiketopiperazines pretrichodermamides D–F (**1**–**3**), together with the known *N*-methylpretrichodermamide B (**4**) and pretrichodermamide С (**5**), were isolated from the lipophilic extract of the marine algae-derived fungus *Penicillium* sp. KMM 4672. The structures of compounds **1**–**5** were determined based on spectroscopic methods. The absolute configuration of pretrichodermamide D (**1**) was established by a combination of modified Mosher′s method, NOESY data, and biogenetic considerations. *N*-Methylpretrichodermamide B (**5**) showed strong cytotoxicity against 22Rv1 human prostate cancer cells resistant to androgen receptor targeted therapies.

## 1. Introduction

**Figure 1:**
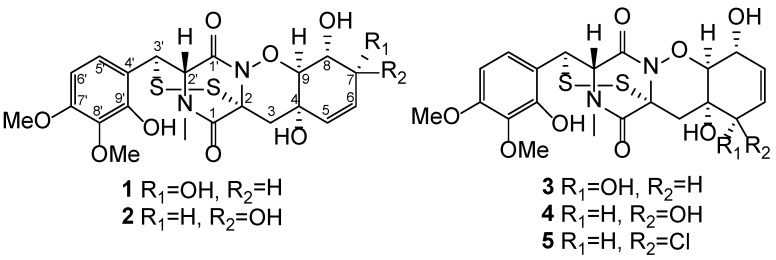
Chemical structures of isolated compounds **1**–**5**.

Epithiodiketopiperazines with 1,2-oxazadecaline moiety are rare in nature. To date, only eleven such compounds have been reported [1,2,3,4]. Structural differences in compounds of this class consist in *N*-methylation, C-4–C-5-epoxidation [5,6] and substituent at C-5. In addition, first mono- and trithioderivatives have recently been reported [3,4]. The fungi of genus *Trichoderma* were producers of most of these alkaloids. However, pretrichodermamide A has also been reported to be synthesized by *Aspergillus* sp. [7], while *N*-methylated compounds methylgliovirin (the first described compound from this class) [5], *N*-methylpretrichodermamide B (adametizine A) [8,9], and pretrichodermamide C (adametizine B) [8,9] have only been isolated from *Penicillium* species. Many oxazadecaline thiodiketopiperazines have been reported and patented as antibiotics, and chloroderivatives have shown cytotoxic activity against murine lymphoma and Jurkat cells with IC_50_ of 2–5 µM [2,8,9,10]. During our ongoing search for structurally novel and bioactive metabolites from marine-derived fungi, we investigated the fungus *Penicillium* sp. KMM 4672 isolated from Vietnamese brown alga *Padina* sp. A chemical study resulted in the isolation and identification of three new 1,2-oxazadecaline epidithiodiketopiperazines pretrichodermamides D–F (**1**–**3**), together with the known pretrichodermamide C (**4**) and *N*-methylpretrichodermamide B (**5**) (Figure 1). Herein, we report the isolation, structure elucidation, and biological assay results of the new compounds **1**–**3** produced by the marine fungus *Penicillium* sp.

## 2. Results

The EtOAc extract of the culture of the fungus was suspended in H_2_O–EtOH (4:1) and successively partitioned with hexane, EtOAc, and *n-*BuOH. The EtOAc portion was subjected to column chromatography over silica gel and by HPLC to yield individual compounds **1**–**5** as white powders.

The molecular formula of compound **1** was determined to be C_21_H_24_N_2_O_9_S_2_ from a HRESIMS peak at *m/z* 511.0857 [M − H]^−^ and was in accordance with ^13^C NMR data. A thorough analysis of the ^1^H and ^13^C NMR data (Table 1) of **1** with DEPT and HSQC techniques revealed the presence of two methoxyls, one N-methyl, one methylene, four sp^2^-methines, and five sp^3^-methines together with two sp^3^-quaternary carbons. The remaining functionalities, corresponding to the carbon signals at δ_C_ 165.4 (C), 164.2 (C), 153.0 (C), 147.6 (C), 135.9 (C), and 116.3 (С), suggested the presence of two amide carbonyl carbons, three oxygenated, and one C-substituted sp^2^-carbons.

A direct comparison of ^1^H and ^13^C NMR spectra of **1** (Table 1) with those of pretrichodermamide C (**4**) [8] (S21–S22, Supplementary data) showed similarities including signals of two methoxyls (δ_H_ 3.68, 3.78; δ_C_ 55.7, 60.2), *N*-methyl (δ_H_ 2.96; δ_C_ 32.6), one phenolic hydroxyl group (δ_H_ 9.43), two aromatic methine (δ_H_ 6.55, 7.32; δ_C_ 103.3, 122.6), and two amide carbonyls (δ_C_ 164.2, 165.4), suggesting that **1** has a framework similar to that of 4.

The HMBC correlations from both H-3 (δ_H_ 2.17, 2.33) to C-4 (δ_C_ 66.9), C-5 (δ_C_ 133.8), and C-9 (δ_C_ 81.9), from 4-OH (δ_H_ 5.26) to C-3 (δ_C_ 38.4), C-4, C-5, and C-9, from H-9 (δ_H_ 4.12) to C-8 (δ_C_ 66.2), and from H-7 (δ_H_ 4.03) to C-5, C-6 (δ_C_ 127.4), and C-8 established the cyclohexene ring with a C-5–C-6 double bond placement. The location of secondary hydroxyl groups at C-7 and C-8 was proven by HMBC correlations from 7-OH (δ_H_ 4.89) to C-7 and from 8-OH (δ_H_ 4.35) to C-8. The planar structure of **1** was thus elucidated.

**Figure 2:**
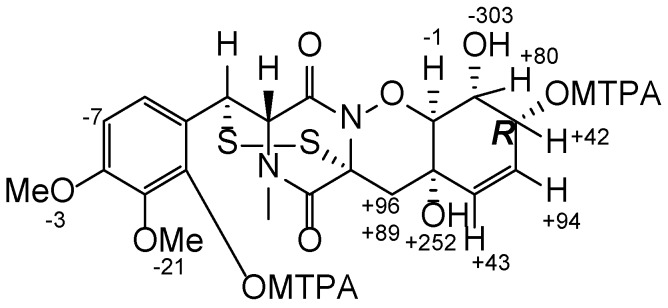
∆δ (δ_S_−δ_R_) values (in Hz) for the MTPA ester of **1**.

**Figure 3:**
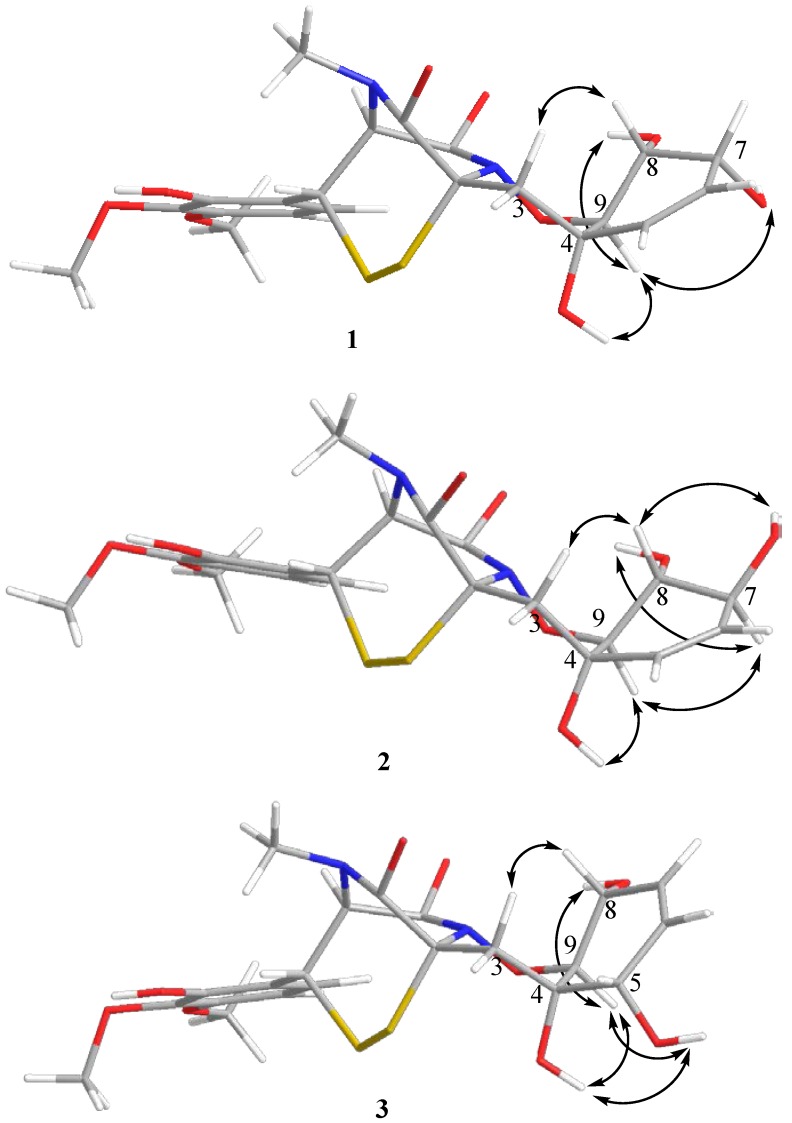
Energy-minimized 3D models of **1**–**3** with selected ROESY correlations.

Esterification of the C-7 and C-9′ hydroxy moieties of **1** with (*R*)- and (*S*)-MTPA chloride afforded the (*S*)- and (*R*)-bis-MTPA-esters, respectively. The observed chemical shift differences Δδ (δ_S_ − δ_R_) (Figure 2) indicated 7*R* configuration [11]. The absolute configurations of the remaining stereocentres in cyclohexene ring were established as 4*S*, 8*R*, 9*S*, the same as in adametizine B (pretrichodermamide C) and adametizine A (*N*-methylpretrichodermamide B) (Figure 3) based on the ROESY-correlations 7-OH with H-9, and H-9 with 4-OH and 8-OH, together with the coupling constants ^3^*J*_H8-H9_ (9.4 Hz) and ^3^*J*_H7-H8_ (4.6 Hz), which were in accordance with calculated dihedral angles (177° and 46°, respectively). The absolute configurations at C-2, C-2′, and C-3′ were assigned to be the same as known adametizine A and adametizine B on the basis of the similarity of C-2, C-2′, and C-3′ chemical shifts for these biogenetically related compounds [9]. Compound **1** was named pretrichodermamide D.

The molecular formula of compound **2** was determined as C_21_H_24_N_2_O_9_S_2_, the same as **1**, by a HRESIMS peak at *m/z* 511.0869 [M − H]^−^ and by ^13^C NMR analysis. The general features of the ^1^H and ^13^C NMR spectra (Table 1) of **2** resembled those of **1** with the exception of the proton and carbon signals at C-7 and C-8. The HMBC correlations from H-7 (δ_H_ 3.96) to C-6 (δ_C_ 129.7) and C-8 (δ_C_ 71.0), from H-9 (δ_H_ 3.83) to C-4 (δ_C_ 67.0) and C-8, and from 4-OH (δ_H_ 5.29) to C-3 (δ_C_ 39.0), C-5 (δ_C_ 131.8), and C-9 (δ_C_ 83.4) proved that the planar structure of **2** was identical with pretrichodermamide D (**1**). The vicinal coupling constant *J*_H7-H8_ (7.7 Hz) and *J*_H8-H9_ (10.7 Hz) according to Karplus equation (calculated dihedral angles are 168° and 174°, respectively) indicated the axial stereolocations of H-7, H-8 (δ_H_ 3.56), and H-9. These relative configurations were additionally proved by ROESY’s (Figure 3) H-7 with 8-OH (δ_H_ 4.64) and H-9. The absolute stereoconfigurations of **2** were assigned as for pretrichodermamide D (**1**) according to biogenetic considerations. Thus, compound **2** was determined as the C-7 epimer of pretrichodermamide D and named pretrichodermamide E.

The molecular formula of compound **3** was determined as C_21_H_24_N_2_O_9_S_2_ (the same as **1** and **2**) on the basis of HRESIMS and ^13^C NMR spectra. The NMR data for this compound were very similar to those obtained for pretrichodermamide C (**4**) with the exception of proton and carbon signals at C-3, C-4, C-5, C-6, and C-9. The HMBC correlations from H-5 (δ_H_ 3.69) to C-3 (δ_C_ 35.6), C-4 (δ_C_ 67.4), C-6 (δ_C_ 126.9), and C-7 (δ_C_ 131.2), from H-8 (δ_H_ 4.16) to C-7 and C-9 (δ_C_ 83.5), and from H-9 (δ_H_ 3.97) to C-4 and C-8 (δ_C_ 64.6) established a planar structure of the cyclohexene ring with a double bond between C-6 and C-7. The mutual ROESY correlations (Figure 3) from H-9 to 4-OH (δ_H_ 4.96), 5-OH (δ_H_ 5.19), and 8-OH (δ_H_ 5.15) showed α-orientation of 5-OH and detected **3** as the С-5-epimer of pretrichodermamide C (**4**). The relative configurations of the 1,2-oxazadecaline fragment were additionally suggested by a W-type coupling constant between H-9 (δ_H_ 3.97, dd, 7.3, 1.5) and H-3β (δ_H_ 2.06, dd, 15.4, 1.5). The absolute configurations in compound **3** were proposed based on biogenetic relationships with compounds **1**, **2**, **4**, and **5**. Compound **3** was named pretrichodermamide F.

Besides the new pretrichodermamides D–F (**1**–**3**), the known pretrichodermamide C (**4**) and *N*-methylpretrichodermamide B (**5**) were also isolated from this fungus. For the first time these compounds were found in Egyptian hyper saline lake fungus *Penicillium* sp. [8] and were later isolated from sponge-derived *Penicillium adametzioides* and published as new adametizines A and B, respectively. The absolute stereochemistry for adametizines were determined based on X-ray and ECD data [9]. The structures of **4** and **5** were established on the basis of 1D and 2D NMR data and high resolution ESIMS analysis (Supplementary data S20–S26). The absolute structures of compounds **4** and **5** were determined the same as for adametizines B and A, respectively, based on identity of their ECD spectra.

In a next step, we investigated the effects of compounds **1**–**5** on viability and the apoptosis induction of human prostate cancer cells. It should be noted that, in a recently published study, *N*-methylpretrichodermamide B did not show any cytotoxic effect against a number of different cancer cells up to 10 µM [9]. MTT assays revealed *N*-methylpretrichodermamide B (**5**) to be highly cytotoxic in 22Rv1, PC-3, and LNCaP cells with IC_50_ 0.51, 5.11, and 1.76 µM, respectively, while revealed IC_50_s of 0.013, 0.015, and 0.004 µM were determined for docetaxel (positive control). Remarkably, **5** induces apoptosis in human prostate cancer 22Rv1 cells (31.3% ± 8.2% apoptosis after treatment with 1 µM for 48 h), which are highly resistant to androgen receptor (AR)-targeted therapies due to a loss of the ligand-binding domain of the AR receptor [12]. Сompounds **1**–**4** did not exhibit cytotoxic activity against human prostate cancer cells at concentrations up to 100 µM. No significant effect on cell cycle progression was observed for any of the compounds at concentrations up to 100 µM. 22Rv1 cells are known to be resistant to the hormone therapy due to the presence of androgen receptor splice variant AR-V7, while LNCaP cells bearing *w*/*t* AR are sensitive to the hormone deprivation [12]. Remarkably, **5** was mostly active in AR-V7-positive 22Rv1 cells with IC_50_ at nanomolar concentrations (MTT test). In addition, the effect of compounds **1**–**5** was tested on non-malignant murine cells (splenocytes and erythrocytes). *N*-methylpretrichodermamide B (**5**) did not show hemolitic activity up to 100 µM and was cytotoxic for splenocytes only at high doses (ED_50_ 62.1 µM).

## 3. Materials and Methods

### 3.1. General Experimental Procedures

Optical rotations were measured on a Perkin-Elmer 343 polarimeter (Perkin Elmer, Waltham, MA, USA). UV spectra were recorded on a Specord UV VIS spectrometer (Carl Zeiss, Jena, Germany) in MeOH. IR spectra were determined on a Specord M 82 (Carl Zeiss, Jena, Germany) in CHCI_3_. NMR spectra were recorded in DMSO-d_6_ on a Bruker DPX-500 (Bruker BioSpin GmbH, Rheinstetten, Germany) (500.13/125.77 MHz) and Bruker DRX-700 (Bruker BioSpin GmbH, Rheinstetten, Germany) (700.00/176.04 MHz) spectrometer, using TMS as an internal standard. HRESIMS spectra were measured on an Agilent 6510 Q-TOF LC mass spectrometer (Agilent Technologies, Santa Clara, CA, USA).

Low pressure liquid column chromatography was performed using silica gel (50/100 μm, Imid, Russia). Plates (4.5 × 6.0 cm) precoated with silica gel (5–17 μm, Imid) were used for thin layer chromatography. Preparative HPLC was carried out on a Shimadzu LC-20 chromatograph (Shimadzu USA Manufacturing, Canby, OR, USA) using a YMC ODS-AM (YMC Co., Ishikawa, Japan) (5 µm, 10 × 250 mm) and YMC SIL (YMC Co., Ishikawa, Japan) (5 µm, 10 × 250 mm) columns with an Shimadzu RID-20A refractometer (Shimadzu Corporation, Kyoto, Japan).

The energy-minimized structure models for **1**–**3** have been calculated based on crystallographic data (CCDC 1040973) for adametizine A (*N*-methylpretrichodermamide, compound **5**) [9] by the MM2 force field calculation method using ChemBio3D Ultra 12.0, CambridgeSoft Corporation (Cambridge, MA, USA).

### 3.2. Fungal Strain

The strain was isolated from brown algae *Padina* sp. (South China Sea, Vietnam) by the plating method using malt extract agar and identified on the basis of morphological and molecular features. For DNA extraction, the culture was grown on malt extract agar under 25 °C for 7 d. DNA extraction was performed by HiPurATM Plant DNA Isolation kit (CTAB Method) (HiMedia Laboratories Pvt. Ltd., Mumbai, India) according to the manufacturer′s instructions. Fragments containing the ITS regions were amplified using primers ITS1 and ITS4 [13]. Amplification of the partial calmodulin gene was performed using Cmd5 and Cmd6 primers [14]. The newly obtained sequences were checked visually and compared to available sequences of GenBank by using BLAST-n. According to BLAST analysis of the ITS1-5.8S-ITS2 and partial calmodulin datasets, the strain *Penicillium* sp. KMM 4672 is related to *P. citrinum*-group and displays the most similarity with *P. steckii* (99% and 97%, respectively). The sequences were deposited in GenBank nucleotide sequence database under KU 695807 and KU 695808. The strain was deposited in the Collection of Marine Microorganisms under the code KMM 4672.

### 3.3. Cultivation of Fungus

The fungus was grown stationary at 22 °С for three weeks in 60 × 500 mL Erlenmeyer flasks, each containing 60 g of the solid nutrient medium of the following composition: rice (20.0 g), yeast extract (20.0 mg), KH_2_PO_4_ (10 mg), and natural sea water (40 mL).

### 3.4. Extraction and Isolation

The fungal mycelia with the medium were extracted for 24 h with 12 L of EtOAc. Evaporation of the solvent under reduced pressure yielded a brown oil (9.2 g), to which 250 mL of H_2_O–EtOH (4:1) was added, and the combination was thoroughly mixed to yield a suspension. It was extracted successively with hexane (150 mL × 2), EtOAc (150 mL × 2) and *n-*BuOH (150 mL × 2). The EtOAc fraction was concentrated in vacuo to give a residue (6.0 g), which was separated on a silica gel column (30 × 3cm) eluted with a hexane-EtOAc gradient (1:0–0:1). The hexane-EtOAc fraction PS-101-64 (65:35, 210 mg) was purified by RP HPLC on a YMC ODS-AM column eluting with MeOH–H_2_O (65:35) to yield **5** (170 mg). The hexane-EtOAc fraction PS-101-87 (50:50, 73 mg) was separated by RP HPLC on a YMC ODS-AM column eluting with MeOH–H_2_O (65:35) to yield the **4** (16 mg), **1** (4.6 mg) and PS-103-4 fractions (7.8 mg). The PS-103-4 fraction was purified by HPLC on a YMC Sil column eluting with CHCl_3_–MeOH (95:5) to yield **2** (3.4 mg) and **3** (3.2 mg).

Pretrichodermamide D (**1**): white powder; ${\left[\alpha \right]}_{D}^{20}$ −205 (*c* 0.17, MeOH); UV (MeOH) λ_max_ (log ε) 205 (4.54) nm; ECD (0.17 mM, MeOH) λ_max_ (Δε) 218 (−20.33), 258 (−6.98), 301 (+0.83) nm; ^1^H and ^13^C NMR data, see Table 1, Supplementary data S1–S6; HRESIMS *m/z* 511.0859 [M − H]^−^ (calcd for C_21_H_23_N_2_O_9_S_2_, 511.0850).

Pretrichodermamide E (**2**): white powder; ${\left[\alpha \right]}_{D}^{20}$ −85 (*c* 0.4, MeOH); UV (MeOH) λ_max_ (log ε) 205 (4.51) nm; ECD (0.21 mM, MeOH) λ_max_ (Δε) 218 (−17.64), 258 (−6.04), 300 (+0.80) nm; IR (CHCl_3_) *ν*_max_ 3514, 3000, 2842, 1694, 1617, 1509, 1466, 1346, 1274, 1097, 1056, 1029 cm^−1^; ^1^H and ^13^C NMR, see Table 1, Supplementary data S7–S13; HRESIMS *m/z* 511.0869 [M − H]^−^ (calcd for C_21_H_23_N_2_O_9_S_2_, 511.0850).

Pretrichodermamide F (**3**): white powder; ${\left[\alpha \right]}_{D}^{20}$ −114 (*c* 0.4, MeOH); UV (MeOH) λ_max_ (log ε) 206 (4.45) nm; ECD (0.17 mM, MeOH) λ_max_ (Δε) 217 (−23.27), 260 (−6.64), 301 (+0.49) nm; IR (CHCl_3_) *ν*_max_ 3515, 3001, 2842, 1692, 1617, 1509, 1466, 1347, 1277, 1098, 1054, 1036 cm^−1^; ^1^H and ^13^C NMR, see Table 1, Supplementary data S14–S20; HRESIMS *m/z* 535.0815 [M + Na]^+^ (calcd for C_21_H_24_N_2_O_9_S_2_Na, 535.0815).

Pretrichodermamide C (**4**): white powder; ${\left[\alpha \right]}_{D}^{20}$ −166.9 (*c* 0.09, MeOH) [lit. ${\left[\alpha \right]}_{D}^{20}$ −167.0 (*c* 0.12, MeOH)] [8]; UV (MeOH) λ_max_ (log ε) 206 (4.34) nm; ECD (0.20 mM, MeOH) λ_max_ (Δε) 217 (−27.17), 262 (−7.03), 303 (+0.69) nm; ^1^H and ^13^C NMR data, see Supplementary data S21–S22; HRESIMS *m/z* 535.0830 [M + Na]^+^ (calcd for C_21_H_24_N_2_O_9_S_2_Na, 535.0815).

*N*-methylpretrichodermamide B (**5**): white powder; ${\left[\alpha \right]}_{D}^{20}$ −232.0 (*c* 0.14, MeOH) [lit. ${\left[\alpha \right]}_{D}^{20}$ −102.0 (*c* 0.07, MeOH)] [8]; UV (MeOH) λ_max_ (log ε) 205 (4.56) nm; ECD (0.32 mM, MeOH) λ_max_ (Δε) 217 (−42.10), 262 (−9.18), 303 (+0.87) nm; ^1^H and ^13^C NMR data, see Supplementary data S23–S26; HRESIMS *m/z* 553.0479 [M + Na]^+^ (calcd for C_21_H_23_N_2_O_8_S_2_ClNa, 553.0477).

### 3.5. Preparation of (S)-MTPA and (R)-MTPA Esters of Pretrichodermamide D (**1**)

4-dimethylaminopyridine (a few crystals) and (*R*)-MTPA-Cl (5 μL) was added to a solution of the pretrichodermamide D (1.8 mg) in pyridine at room temperature and stirred for 4 h. After evaporation of the solvent, the residue was passed through a silica gel column (20% EtOAc-hexane) to afford the (*S*)-MTPA ester (1.0 mg). The (*R*)-MTPA ester (1.2 mg) was prepared in a similar manner using (*S*)-MTPA-Cl.

(*S*)-MTPA ester of **1**: ^1^H NMR (DMSO-*d*_6_, 500.13 MHz) δ: 7.16 (1H, d, *J* = 9.4 Hz, H-6′), 6.04 (1H, s, 4-OH), 5.93 (1H, d, *J* = 9.8 Hz, H-5), 5.80 (1H, dd, *J* = 9.8; 5.2 Hz, H-6), 5.73 (1H, t, *J* = 4.9, H-7), 4.67 (1H, d, *J* = 3.7, 8-OH), 4.30 (1H, dt, *J* = 11.0; 4.5 Hz, H-8), 3.89 (3H, s, 8′-OMe), 3.68 (3H, s, 7′-OMe), 3.68 (3H, s, OMe), 3.55 (3H, s, OMe), 2.90 (3H, s, Me-10′), 2.47 (1H, d, *J* = 15.3 Hz, H_2_-3), 2.38 (1H, d, *J* = 15.3 Hz, H_2_-3), 7.45–7.75 (11H, m, 2Ph, H-5′). The signals of H-2′ and H-3′ overlapped with solvent signals. HRESIMS *m/z* 979.1438 [M + Cl]^–^ (calcd for C_41_H_38_N_2_O_13_S_2_F_6_Cl, 979.1414).

(*R*)-MTPA ester of **1**: ^1^H NMR (DMSO-d_6_, 500.13 MHz) δ: 7.66 (1H, d, *J* = 8.9 Hz, H-5′), 7.17 (1H, d, *J* = 8.9 Hz, H-6′), 5.54 (1H, s, 4-OH), 5.85 (1H, d, *J* = 9.7 Hz, H-5), 5.64 (1H, dd, *J* = 9.7; 5.0 Hz, H-6), 5.65 (1H, t, *J* = 5.0, H-7), 5.27 (1H, d, *J* = 5.0, 8-OH), 4.15 (1H, dt, *J* = 10.3; 5.0 Hz, H-8), 3.89 (3H, s, 8′-OMe), 3.73 (3H, s, 7′-OMe), 3.72 (3H, s, OMe), 3.59 (3H, s, OMe), 2.28 (1H, d, *J* = 15.3 Hz, H_2_-3), 2.21 (1H, d, *J* = 15.3 Hz, H_2_-3), 7.40–7.68 (11H, m, 2Ph). The signals of H-2′, H-3′, and Me-10′ overlapped with solvent signals. HRESIMS *m/z* 979.1430 [M + Cl]^–^ (calcd for C_41_H_38_N_2_O_13_S_2_F_6_Cl, 979.1414).

### 3.6. Cell Culture

The human prostate cancer cells lines 22Rv1, PC-3, and LNCaP were purchased from ATCC. Cell lines were cultured according to the manufacturers instructions in 10% FBS/RPMI media (Invitrogen) with (for LNCaP) or without (for 22Rv1 and PC-3) 1 mM sodium pyruvate (Invitrogen). Cells were continuously kept in culture for a maximum of 3 months and were routinely inspected microscopically for stable phenotype and regularly checked for contamination with mycoplasma. Cell line authentication was performed by DSMZ (Braunschweig, Germany) using highly polymorphic short tandem repeat loci [15].

### 3.7. Cytotoxicity Assay

The in vitro cytotoxicity of individual substances was evaluated using the MTT (3-(4,5-dimethylthiazol-2-yl)-2,5-diphenyltetrazolium bromide) assay, which was performed as previously described [16]. Cytotoxicity against CD-I mouse splenocytes was determined according to Freshney [17]. Docetaxel was used as a reference substance.

### 3.8. Cell Cycle and Apoptosis Induction Analysis

The cell cycle distribution was analyzed by flow cytometry using PI staining as described before with slight modifications [18]. In brief, cells were pre-incubated overnight in 6-well plates (2 × 10^5^ cells/well in 2 mL/well). The medium was changed to fresh medium containing different concentrations of the substances. After 48 h of treatment, cells were harvested with a trypsin-EDTA solution, fixed with 70% EtOH, stained, and analyzed by FACS. The results were quantitatively analyzed using Cell Quest Pro software (Version 5.2.1., BD Bioscience, Bedford, MA, USA). Cells appeared at sub-G1 peak were assumed as apoptotic.

### 3.9. Hemolytic Activity 

The hemolytic activity was evaluated using CD-I mouse erythrocytes as previously described [19,20].

## 4. Conclusions

Three new epidithiodiketopiperazines, named pretrichodermamides **D**–**F** (**1**–**3**) were isolated from the lipophilic extract of marine algae-derived fungus *Penicillium* sp. KMM 4672. Each new compound contains rare 1,2-oxazadecaline moieties [1]. Compounds **1** and **2** are the first isomers at oxazadecaline moiety among the related alkaloids. *N*-methylpretrichodermamide B (**5**), highly cytotoxic in 22Rv1 human prostate cancer cells, is resistant to androgen receptor-targeted therapies. At the same time, *N*-methylpretrichodermamide B was found to be cytotoxic for non-malignant cells (splenocytes and erythrocytes) only at high doses (ED50 62.1 and >100 µM). Therefore, this compound may be a promising candidate for the therapy of human drug-resistant prostate cancer.

## Figures and Tables

**Table 1:** NMR spectroscopic data (DMSO-*d*_6_) for pretrichodermamides D–F (**1**–**3**).

Position	1 ^a^		2 ^b^		3 ^b^	
	δ_С_, mult	δ_H_ (*J* in Hz)	δ_С_, mult	δ_H_ (*J* in Hz)	δ_С_, mult	δ_H_ (*J* in Hz)
1	165.4, C	-	165.5, C	-	165.4, C	-
2	68.0, C	-	67.7, C	-	69.1, C	-
3	38.4, CH_2_	α: 2.17, d (15.3) β: 2.33, d (15.3)	39.0, CH_2_	α: 2.22, d (15.7) β: 2.27, d (15.5)	35.6, CH_2_	α: 1.93, brd (15.5) β: 2.06, dd (15.4, 1.5)
4	66.9, C	-	67.0, C	-	67.4, C	-
5	133.8, CH	5.56, d (10.1)	131.8, CH	5.54, dd (10.1, 2.2)	69.2, CH	3.69, d (5.5)
6	127.4, CH	5.60, dd (10.0, 4.4)	129.7, CH	5.43, dd (10.2, 2.2)	126.9, CH	5.69, ddd (10.0, 5.1, 2.3)
7	65.8, CH	4.03, q (4.5)	72.2, CH	3.96, tt (7.7, 2.2)	131.2, CH	5.56, dd (9.9, 2.5)
8	66.2, CH	3.74, ddd (9.4, 6.6, 4.6)	71.0, CH	3.56, ddd (10.7, 7.7, 5.7)	64.6, CH	4.16, m
9	81.9, CH	4.12, d (9.4)	83.4, CH	3.83, d (10.7)	83.5, CH	3.97, dd (7.1, 1.5)
1′	164.2, C	-	164.4, C	-	163.8, C	-
2′	66.0, CH	4.56, d (2.6)	65.7, CH	4.57, d (2.6)	65.4, CH	4.57, d (2.6)
3′	41.4, CH	4.55, d (2.5)	41.5, CH	4.58, d (2.5)	41.0, CH	4.59, d (2.5)
4′	116.3, C	-	116.3, C	-	116.3, C	-
5′	122.6, CH	7.32, d (8.8)	122.6, CH	7.32, d (8.8)	122.7, CH	7.35, d (8.8)
6′	103.3, CH	6.55, d (8.8)	103.3, CH	6.55, d (8.8)	103.2, CH	6.54, d (8.8)
7′	153.0, C	-	153.0, C	-	152.8, C	-
8′	135.9, C	-	135.9, C	-	135.8, C	-
9′	147.6, C	-	147.5, C	-	147.5, C	-
10′	32.6, CH_3_	2.96, s	32.6, CH_3_	2.96, s	32.5, CH_3_	2.96, s
7′-OMe	55.7, CH_3_	3.78, s	55.6, CH_3_	3.78, s	55.6, CH_3_	3.78, s
8′-OMe	60.2, CH_3_	3.68, s	60.2, CH_3_	3.68, s	60.2, CH_3_	3.68, s
9′-OH	-	9.43, s	-	9.42, s	-	9.38, s
4-OH	-	5.26, s	-	5.29, s	-	4.96, brs
5-OH	-	-	-	-	-	5.19, d (5.6)
7-OH	-	4.89, d (5.4)	-	5.02, d (6.9)	-	
8-OH	-	4.35, d (6.6)	-	4.64, d (5.7)	-	5.15, d (6.7)

^a^ ^1^H NMR and ^13^C NMR spectroscopic data were measured at 500.13 MHz and 125.77 MHz, respectively; ^b^ ^1^H NMR and ^13^C NMR spectroscopic data were measured at 700.00 MHz and 176.04 MHz, respectively.

## Supplementary Materials

^1^H, ^13^C, DEPT, COSY-45, HSQC, HMBC, and ROESY spectra of new compounds **1**–**3**, ^1^H, ^13^C, and DEPT spectra of compounds **4** and **5** are available online at www.mdpi.com/1660-3397/14/6/122/s1.

## References

[1] Mfuh A.M., Zhang Y., Stephens D.E., Vo A.X.T., Arman H.D., Larionov O.V. (2015). Concise total synthesis of trichodermamides A, B, and C enabled by an efficient construction of the 1,2-oxazadecaline core. J. Am. Chem. Soc..

[2] Yamazaki H., Rotinsulu H., Narita R., Takahashi R., Namikoshi M. (2015). Induced production of halogenated epidithiodiketopiperazines by a marine-derived *Trichoderma* cf. *brevicompactum* with sodium halides. J. Nat. Prod..

[3] Yamazaki H., Takahashi O., Murakami K., Namikoshi M. (2015). Induced production of a new unprecedented epitrithiodiketopiperazine, chlorotrithiobrevamide, by a culture of the marine-derived *Trichoderma* cf. *brevicompactum* with dimethyl sulfoxide. Tetrahedron Lett..

[4] Kajula M., Ward J.M., Turpeinen A., Tejesvi M.V., Hokkanen J., Tolonen A., Häkkänen H., Picart P., Ihalainen J., Sahl H.G. (2016). Bridged epipolythiodiketopiperazines from *Penicillium raciborskii*, an endophytic fungus of *Rhododendron tomentosum* Harmaja. J. Nat. Prod..

[5] Miyamoto C., Yokose K., Furumai T., Maruyama H.B. (1982). A new epidithiodiketopiperazine group antibiotic, FA-2097. J. Antibiot..

[6] Stipanovic R.D., Howell C.R. (1982). The structure of gliovirin, a new antibiotic from *Gliocladium virens*. J. Antibiot..

[7] Zhou Y., Debbab A., Mándi A., Wray V., Schulz B., Müller W.E.G., Kassack M., Lin W., Kurtán T., Proksch P. (2013). Alkaloids from the sponge-associated fungus *Aspergillus* sp.. Eur. J. Org. Chem..

[8] Orfali R.S., Aly A.H., Ebrahim W., Abdel-Aziz M.S., Müller W.E.G., Lin W., Daletos G., Proksch P. (2015). Pretrichodermamide C and *N*-methylpretrichodermamide B, two new cytotoxic epidithiodiketopiperazines from hyper saline lake derived *Penicillium* sp.. Phytochem. Lett..

[9] Liu Y., Li X.M., Meng L.H., Jiang W.L., Xu G.M., Huang C.G., Wang B.G. (2015). Bisthiodiketopiperazines and acorane sesquiterpenes produced by the marine-derived fungus *Penicillium adametzioides* AS-53 on different culture media. J. Nat. Prod..

[10] Yokose K., Nakayama N., Miyamoto C., Furumai T., Maruyama H.B., Stipanovic R.D., Howell C.R. (1984). Structure of Fa-2097, a new member of the dioxopiperazine antibiotics. J. Antibiot..

[11] Kusumi T., Ooi T., Ohkubo Y., Yabuuchi T. (2006). The modified Mosher’s method and the sulfoximine method. Bull. Chem. Soc. Jpn..

[12] Liu C., Lou W., Zhu Y., Nadiminty N., Schwartz C.T., Evans C.P., Gao A.C. (2014). Niclosamide inhibits androgen receptor variants expression and overcomes enzalutamide resistance in castration-resistant prostate cancer. Clin. Cancer Res..

[13] White T.J., Bruns T., Lee S., Taylor J.W., Innis M.A., Gelfand D.H., Sninsky J.J., White T.J. (1990). Amplificaion and direct sequencing of fungal ribosomal RNA genes for phylogenetics. PCR Protocols: A Guide to Methods and Applications.

[14] Hong S.B., Go S.J., Shin H.D., Frisvad J.C., Samson R.A. (2005). Polyphasic taxonomy of *Aspergillus fumigatus* and related species. Mycologia.

[15] Dyshlovoy S.A., Menchinskaya E.S., Venz S., Rast S., Amann K., Hauschild J., Otte K., Kalinin V.I., Silchenko A.S., Avilov S.A. (2016). The marine triterpene glycoside frondoside A exhibits activity in vitro and in vivo in prostate cancer. Int. J. Cancer.

[16] Dyshlovoy S.A., Venz S., Shubina L.K., Fedorov S.N., Walther R., Jacobsen C., Stonik V.A., Bokemeyer C., Balabanov S., Honecker F. (2014). Activity of aaptamine and two derivatives, demethyloxyaaptamine and isoaaptamine, in cisplatin-resistant germ cell cancer. J. Proteom..

[17] Freshney R.I. (1994). Culture of Animal Cells: A Manual of Basic Technique.

[18] Dyshlovoy S.A., Hauschild J., Amann K., Tabakmakher K.M., Venz S., Walther R., Guzii A.G., Makarieva T.N., Shubina L.K., Fedorov S.N. (2015). Marine alkaloid monanchocidin a overcomes drug resistance by induction of autophagy and lysosomal membrane permeabilization. Oncotarget.

[19] Malagoli D. (2007). A full-length protocol to test hemolytic activity of palytoxin on human erythrocytes. Invertebr. Surviv. J..

[20] Taniyama S., Arakawa O., Terada M., Nishio S., Takatani T., Mahmud Y., Noguchi T. (2003). *Ostreopsis* sp., a possible origin of palytoxin (PTX) in parrotfish *Scarus ovifrons*. Toxicon.

