# Supplementary material for: Pretrichodermamides D–F from a Marine Algicolous Fungus Penicillium sp. KMM 4672
Source: Mar Drugs. 2016 Jun 27;14(7):122. doi: 10.3390/md14070122 (PMC4962012; doi:10.3390/md14070122)
Supplement: Supplementary file 1 [file marinedrugs-14-00122-s001.pdf]

Supplementary Materials: Pretrichodermamides D–F from a Marine Algicolous Fungus *Penicillium* sp. KMM 4672

Anton N. Yurchenko [1,*], Olga F. Smetanina [1], Elena V. Ivanets [2], Anatoly I. Kalinovsky [1], Yuliya V. Khudyakova [1], Natalya N. Kirichuk [1], Roman S. Popov [1], Carsten Bokemeyer [3], Gunhild von Amsberg [3], Ekaterina A. Chingizova [1], Shamil Sh. Afiyatullov [1] and Sergey A. Dyshlovoy [1,2,3]

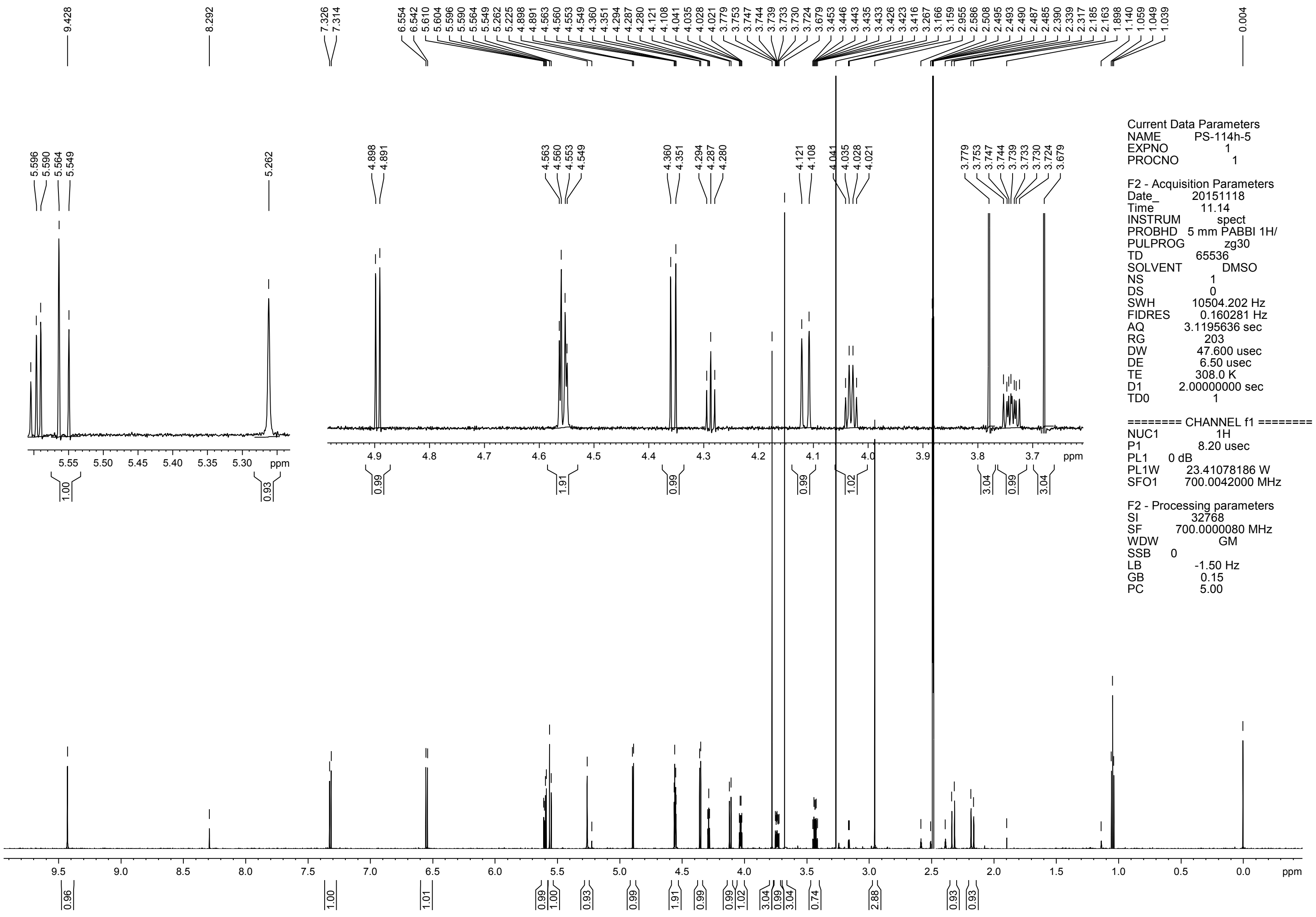

Figure S1. ^{1}H NMR (700 MHz, DMSO-d$_6$) spectrum of pretrichodermamide D (**1**).

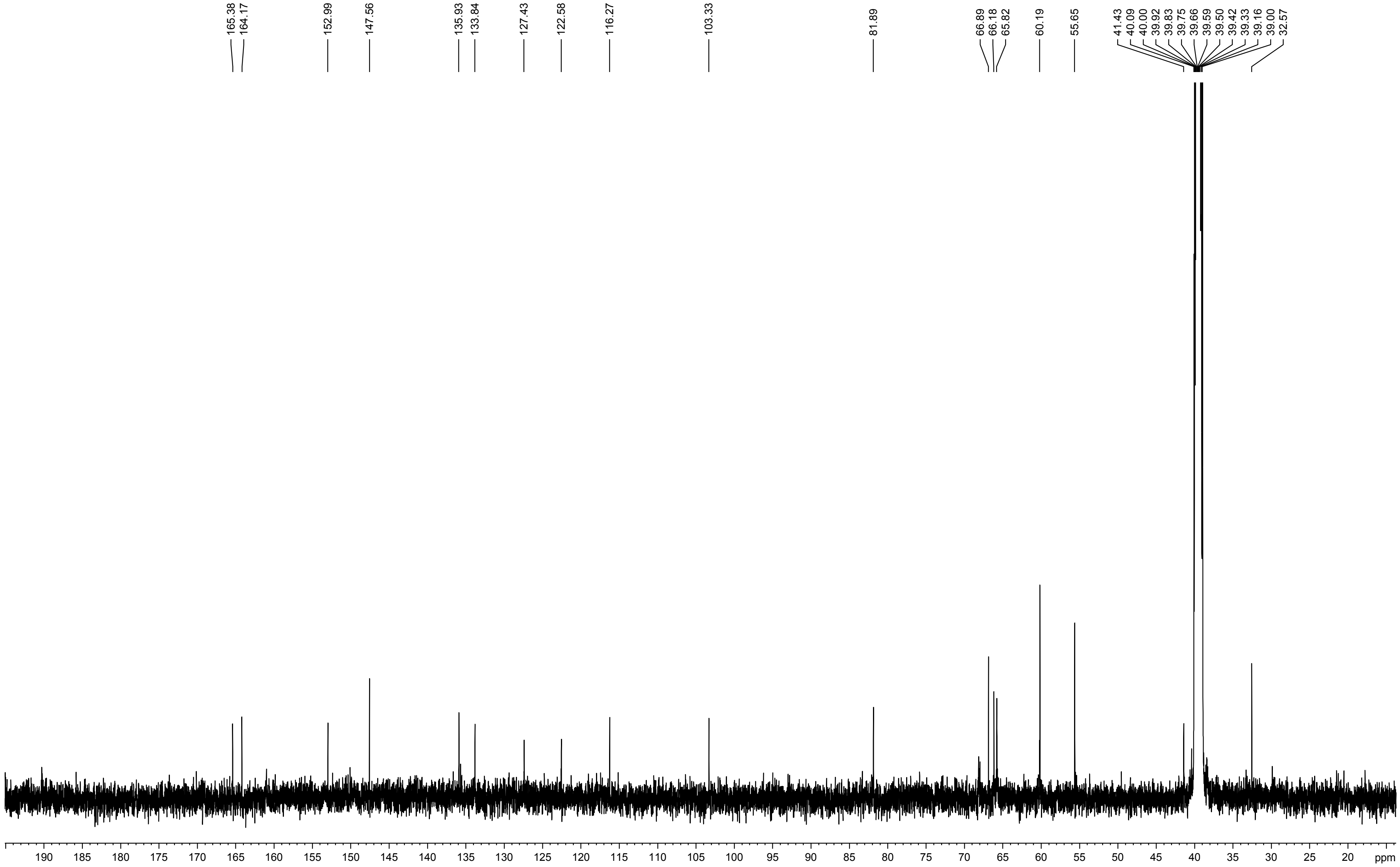

Figure S2. ^{13}C NMR (125 MHz, DMSO-d_6) spectrum of pretrichodermamide D (**1**).

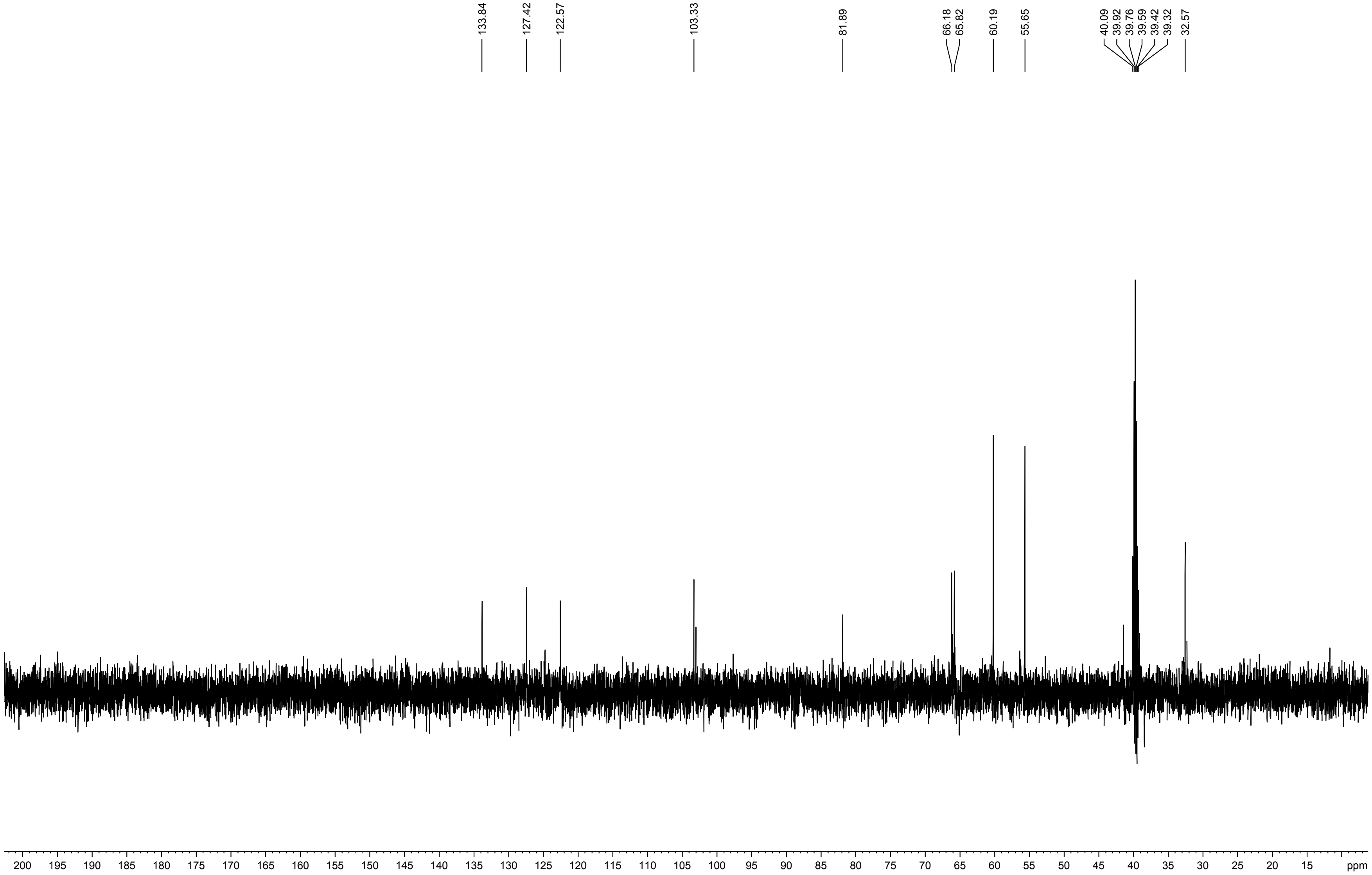

Figure S3. DEPT-135 (125 MHz, DMSO-d$_6$) spectrum of pretrichodermamide D (**1**).

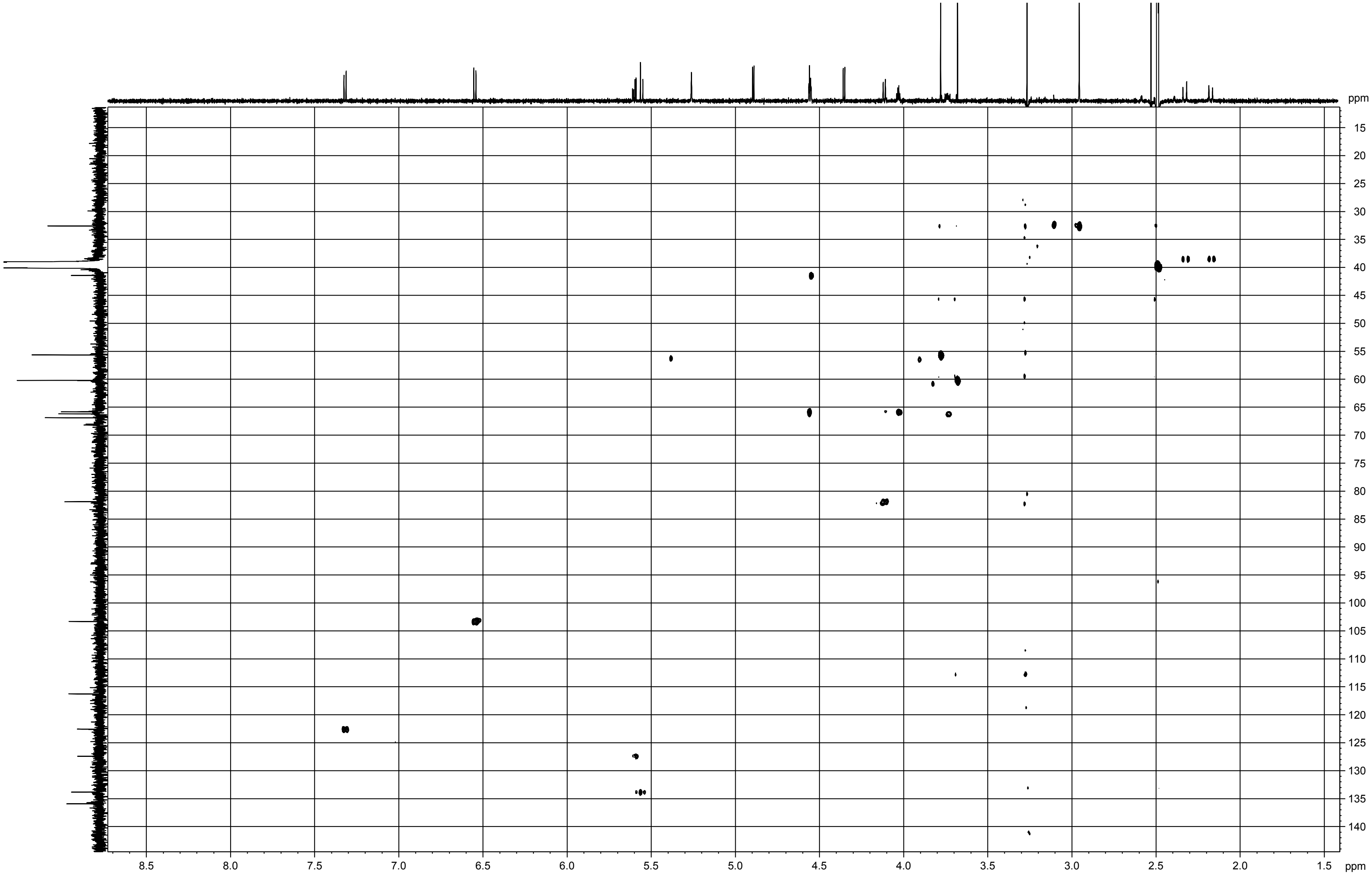

Figure S4. HSQC (500/125 MHz, DMSO-d$_6$) spectrum of pretrichodermamide D (**1**).

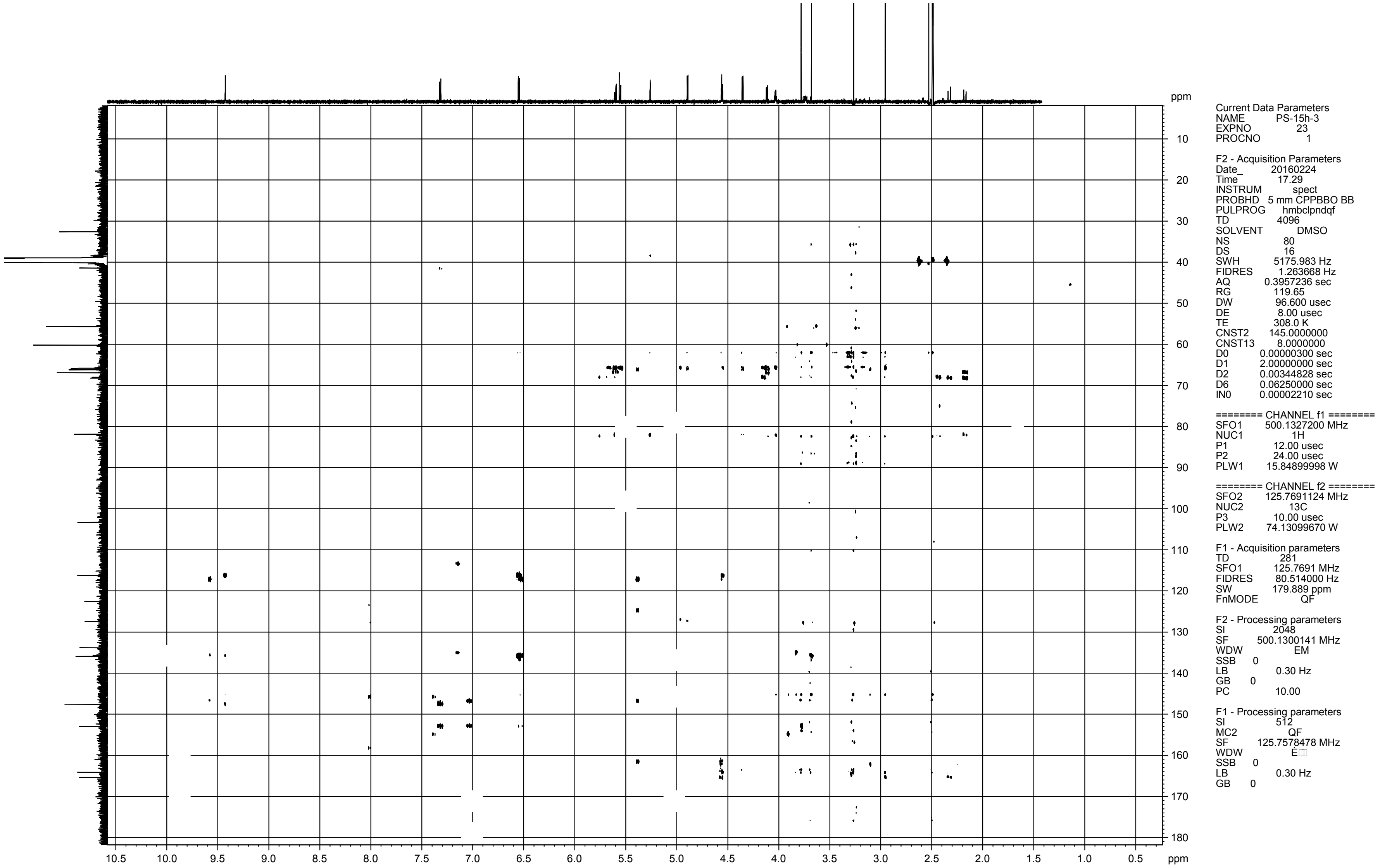

Figure S5. HMBC (500 MHz, DMSO-d_6) spectrum of pretrichodermamide D (**1**).

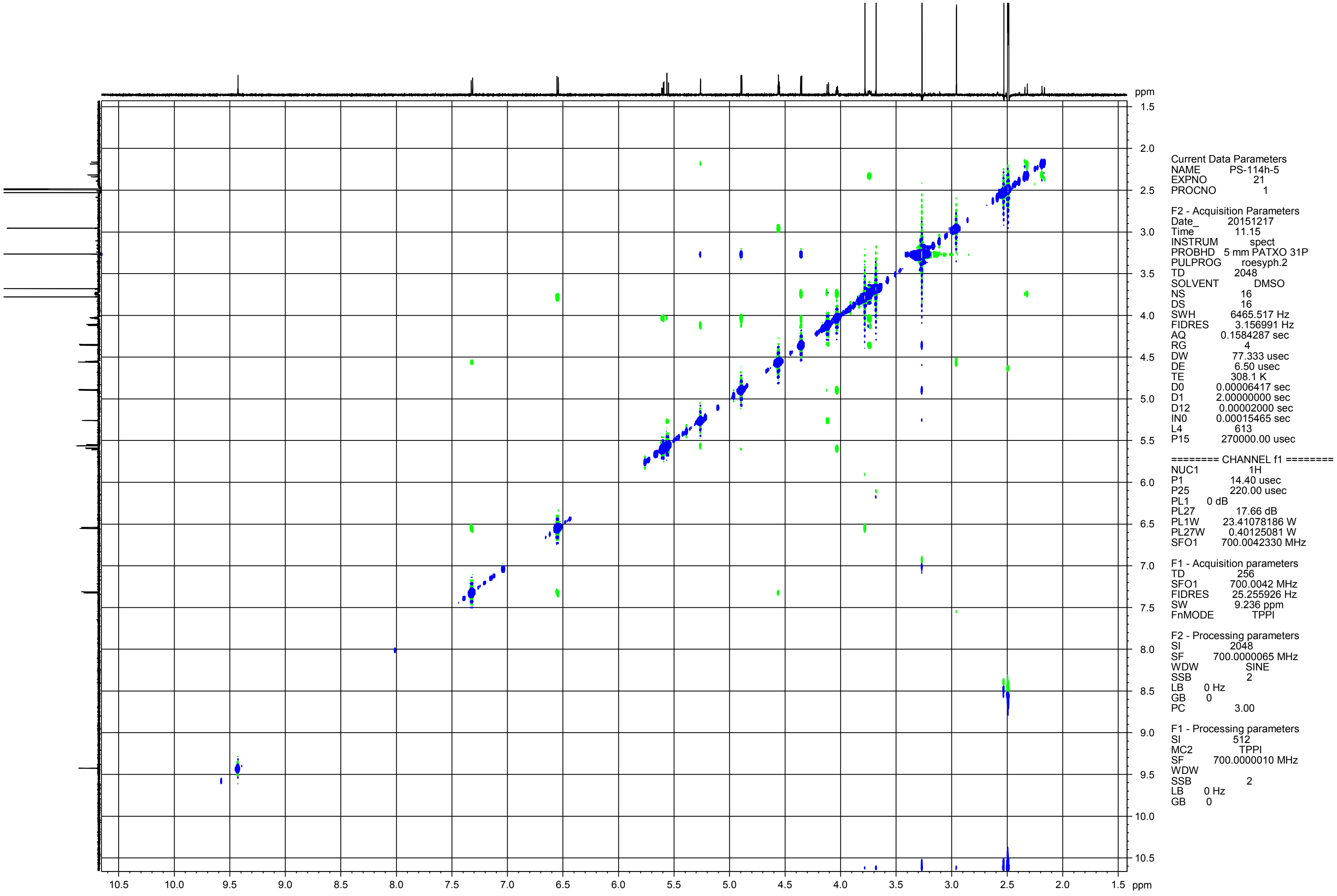

Figure S6. ROESY (700 MHz, DMSO-d$_6$) spectrum of pretrichodermamide D (**1**).

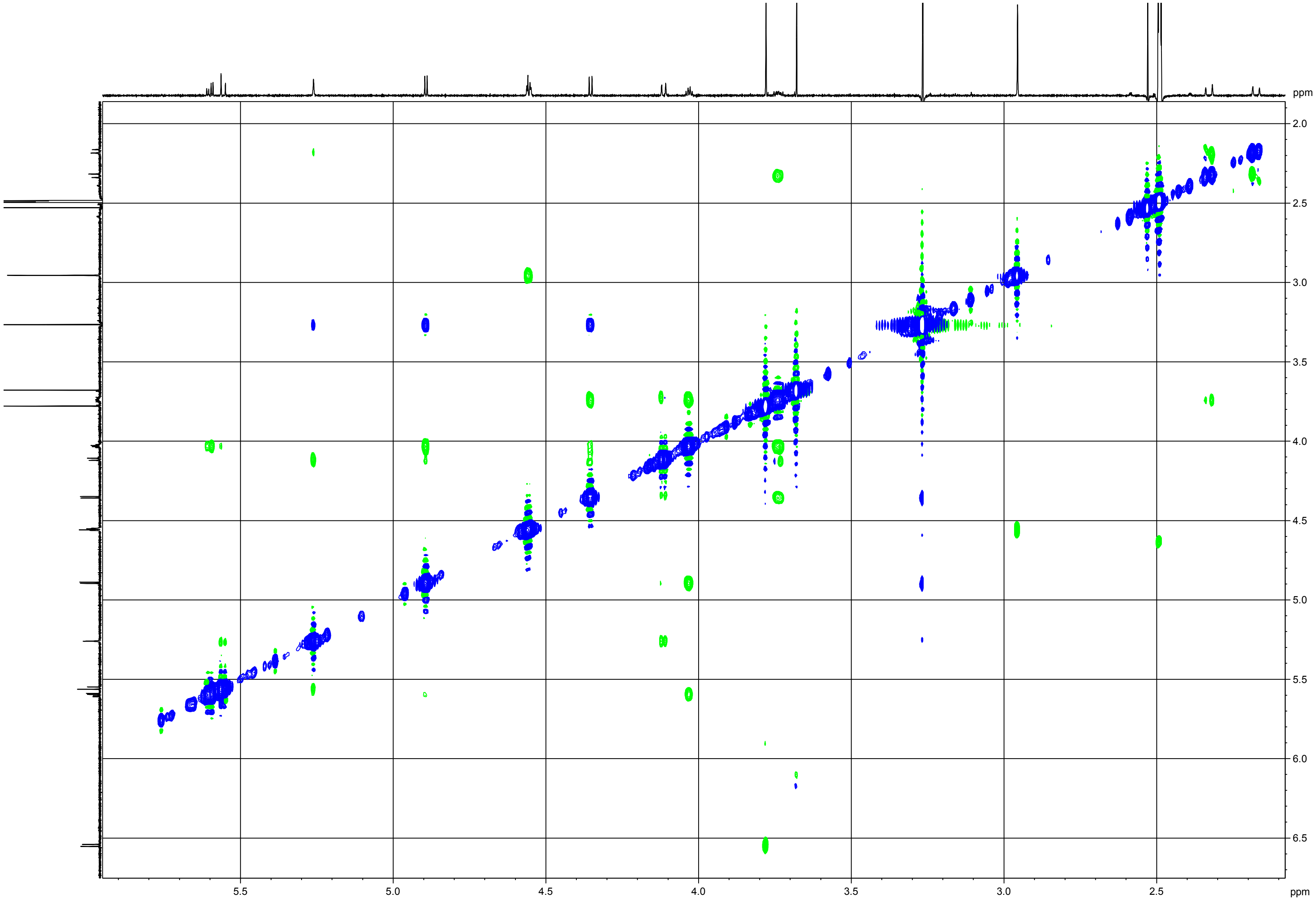

Figure S6 (continuation). ROESY (700 MHz, DMSO-d_6) spectrum of pretrichodermamide D (**1**).

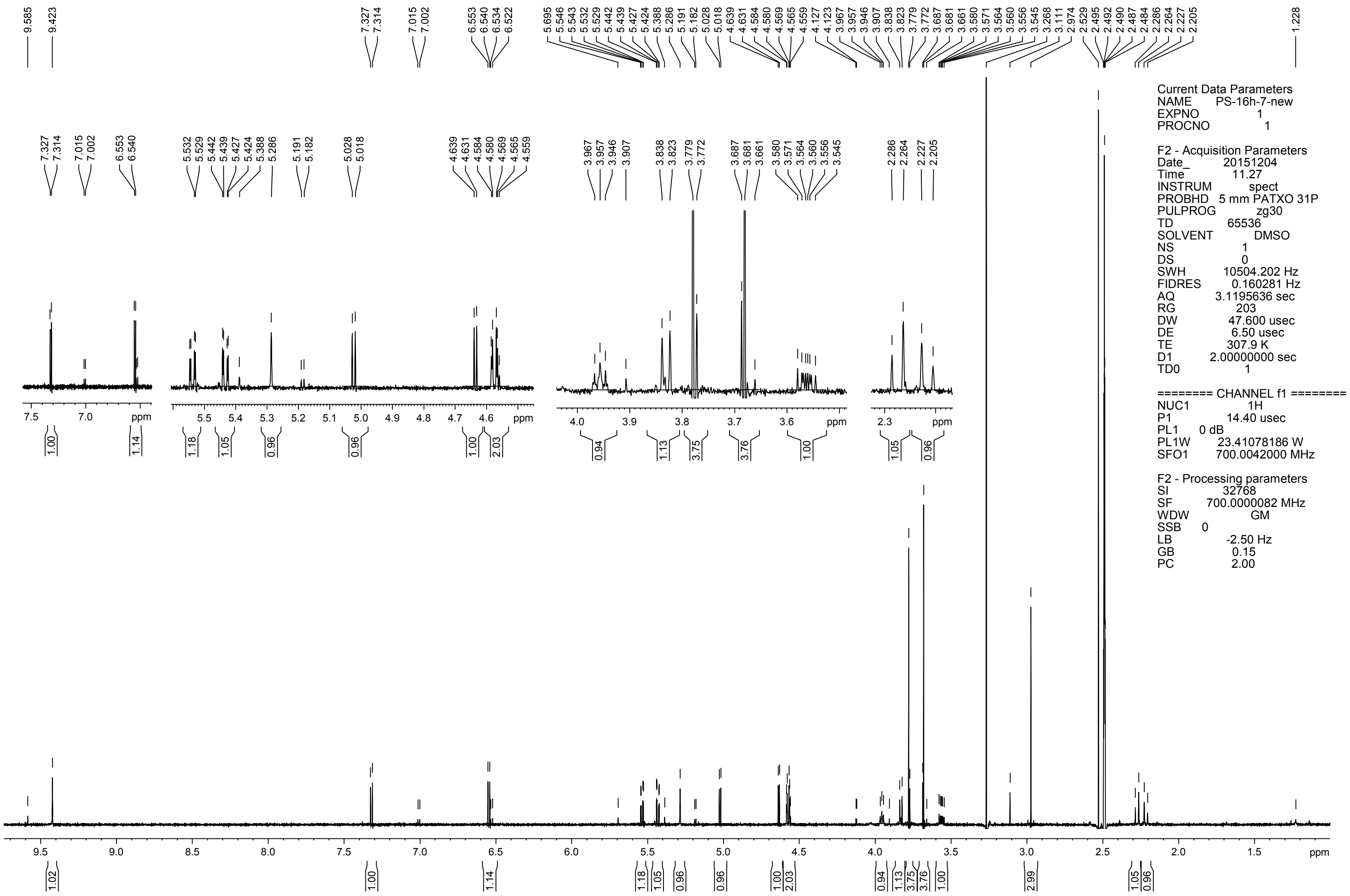

Figure S7. ^{1}H NMR (700 MHz, DMSO-d$_6$) spectrum of pretrichodermamide E (**2**).

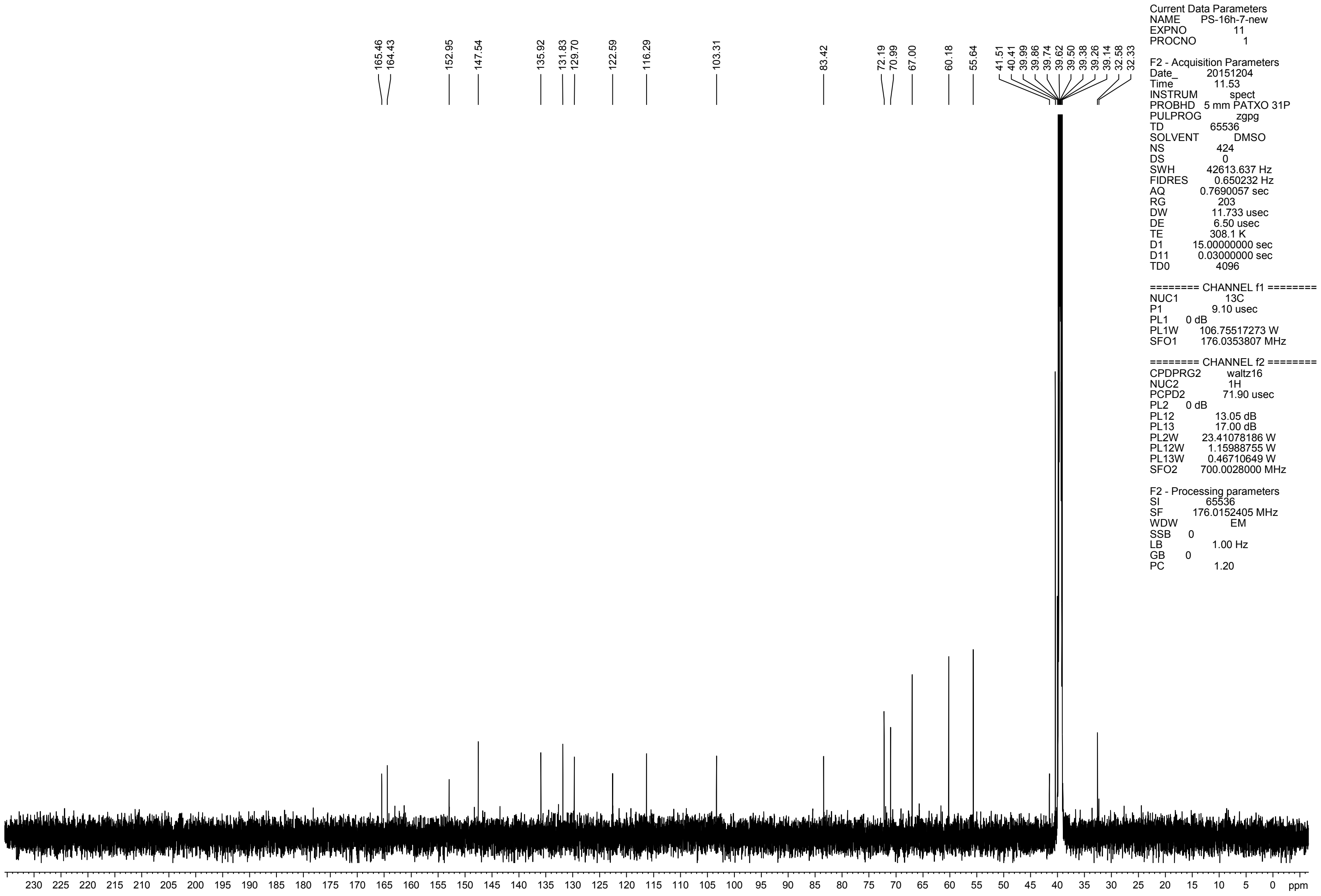

Figure S8. ^{13}C NMR (176 MHz, DMSO-d_6) spectrum of pretrichodermamide E (**2**).

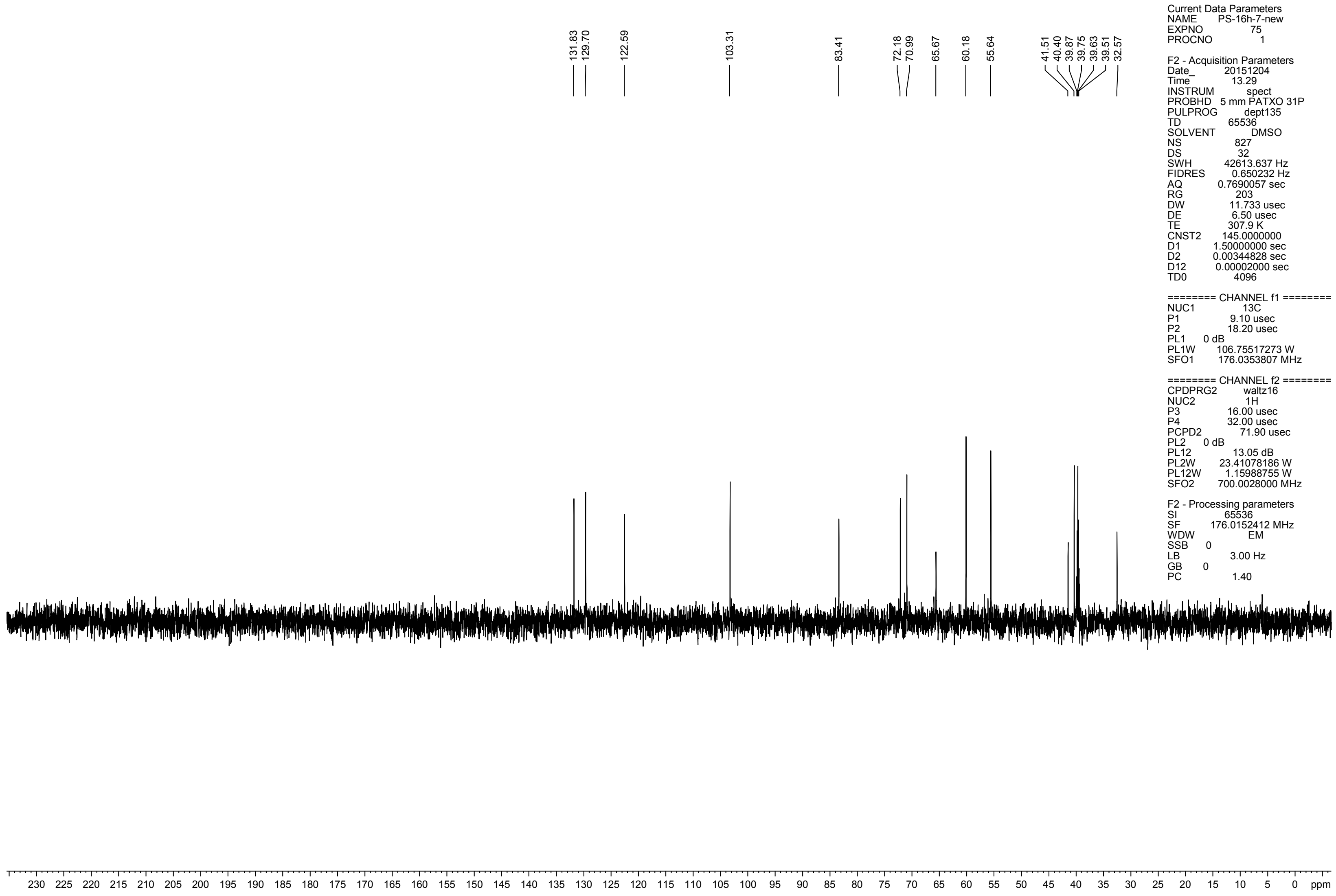

Figure S9. DEPT-135 (176 MHz, DMSO-d6) spectrum of pretrichodermamide E (**2**).

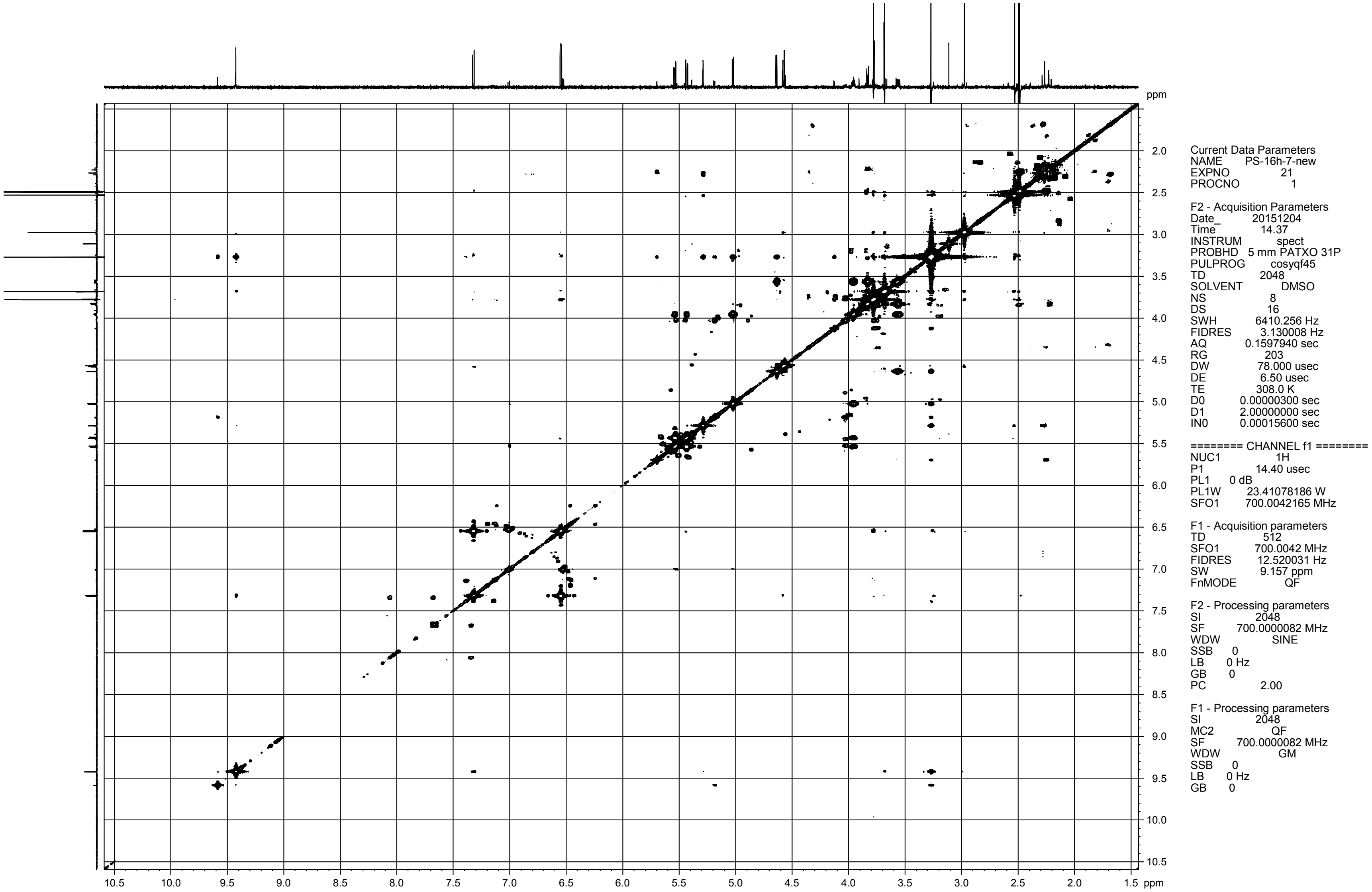

Figure S10. ^{1}H-^{1}H COSY (700 MHz, DMSO-d_6) spectrum of pretrichodermamide E (**2**).

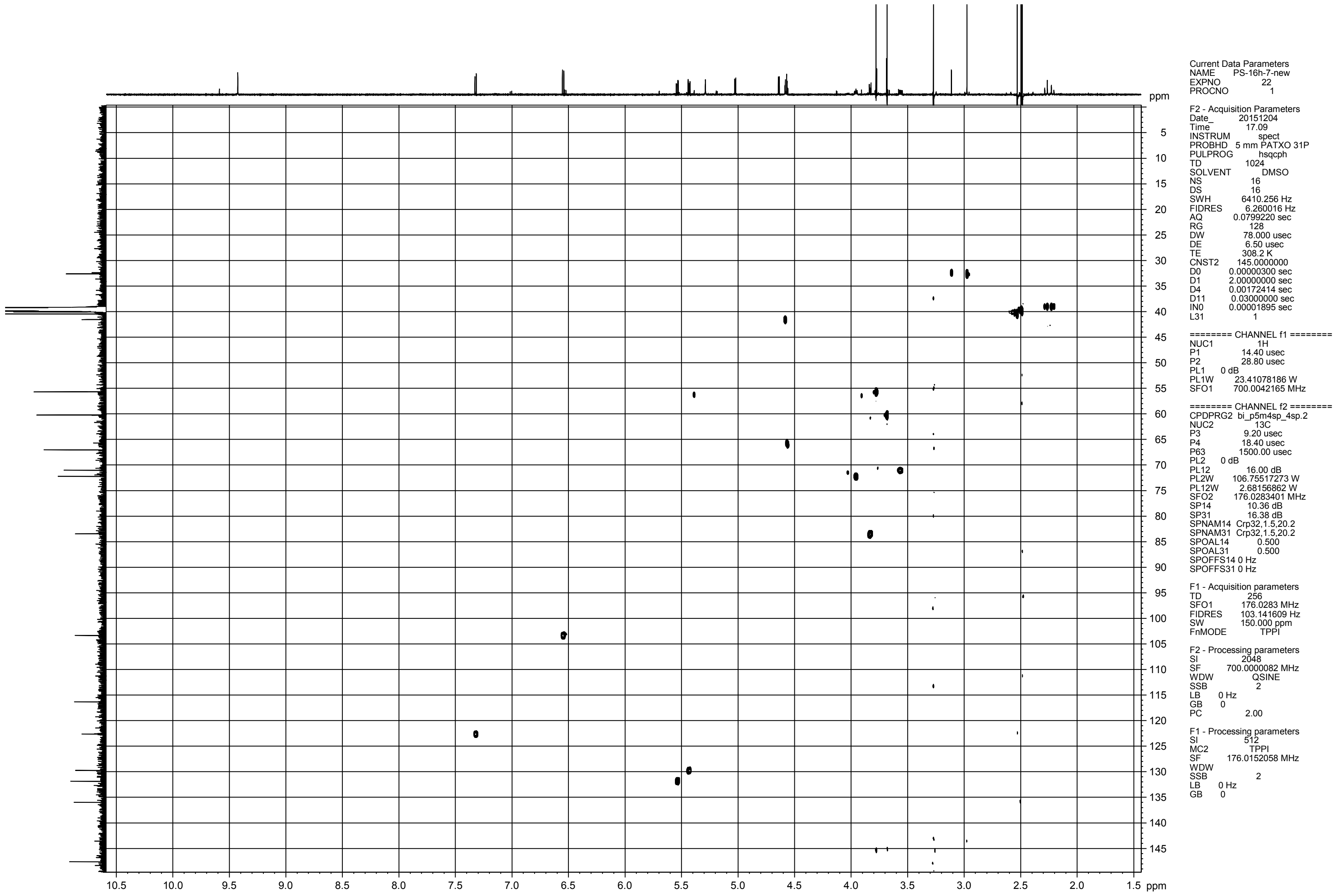

Figure S11. HSQC (700 MHz, DMSO-d₆) spectrum of pretrichodermamide E (**2**).

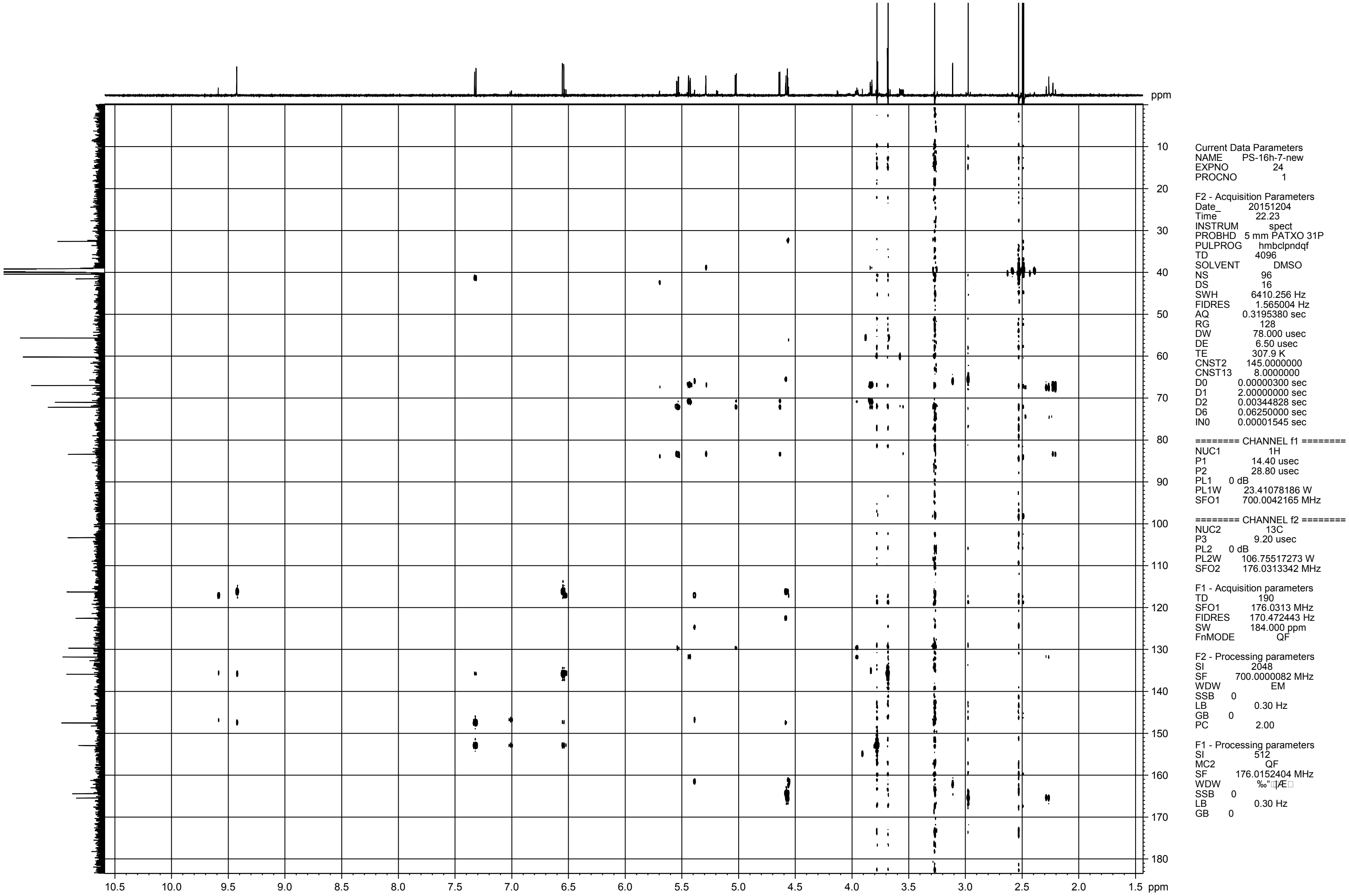

Figure S12. HMBC (700 MHz, DMSO-d6) spectrum of pretrichodermamide E (**2**).

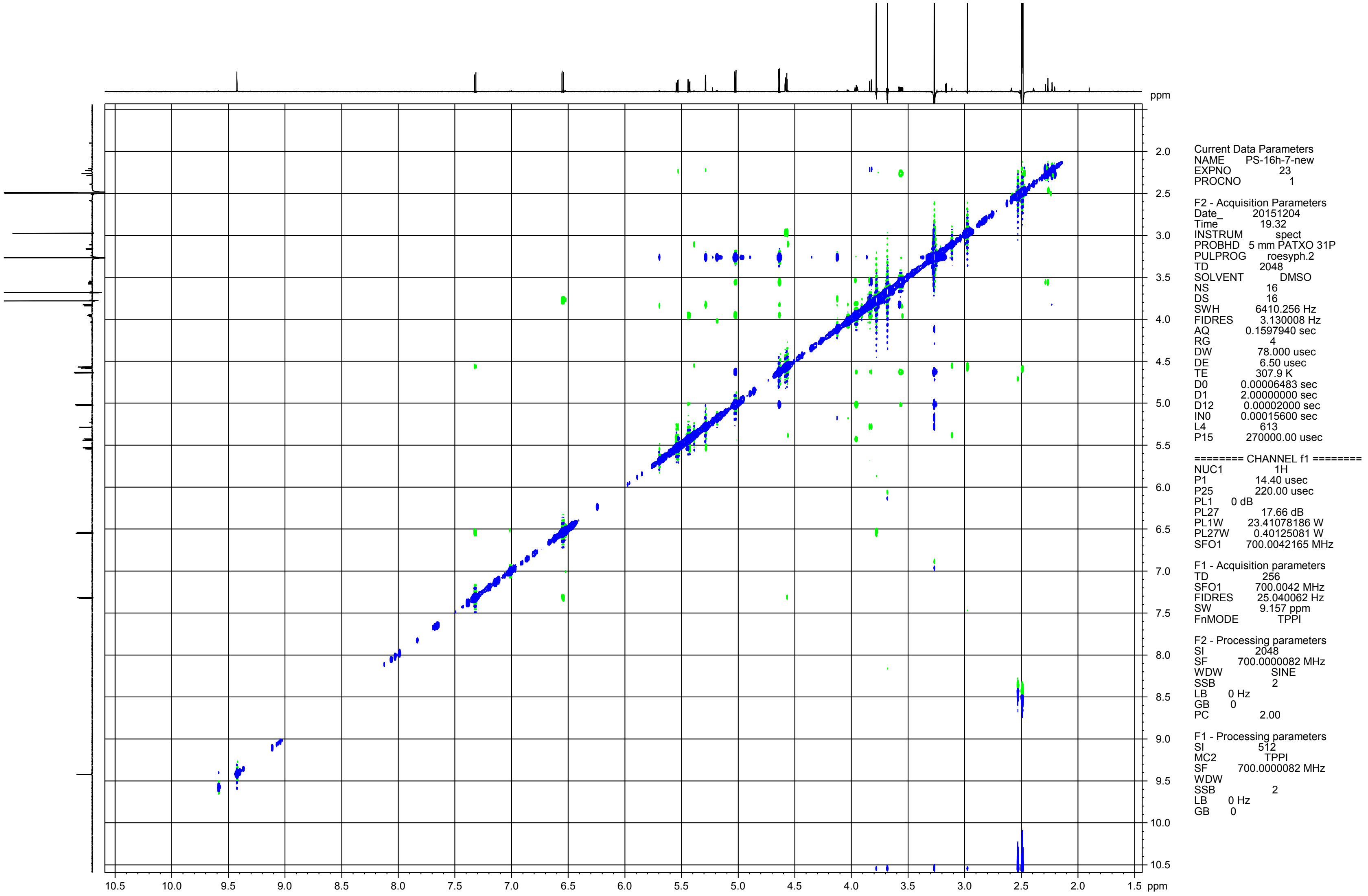

Figure S13. ROESY (700 MHz, DMSO-d$_6$) spectrum of pretrichodermamide E (**2**).

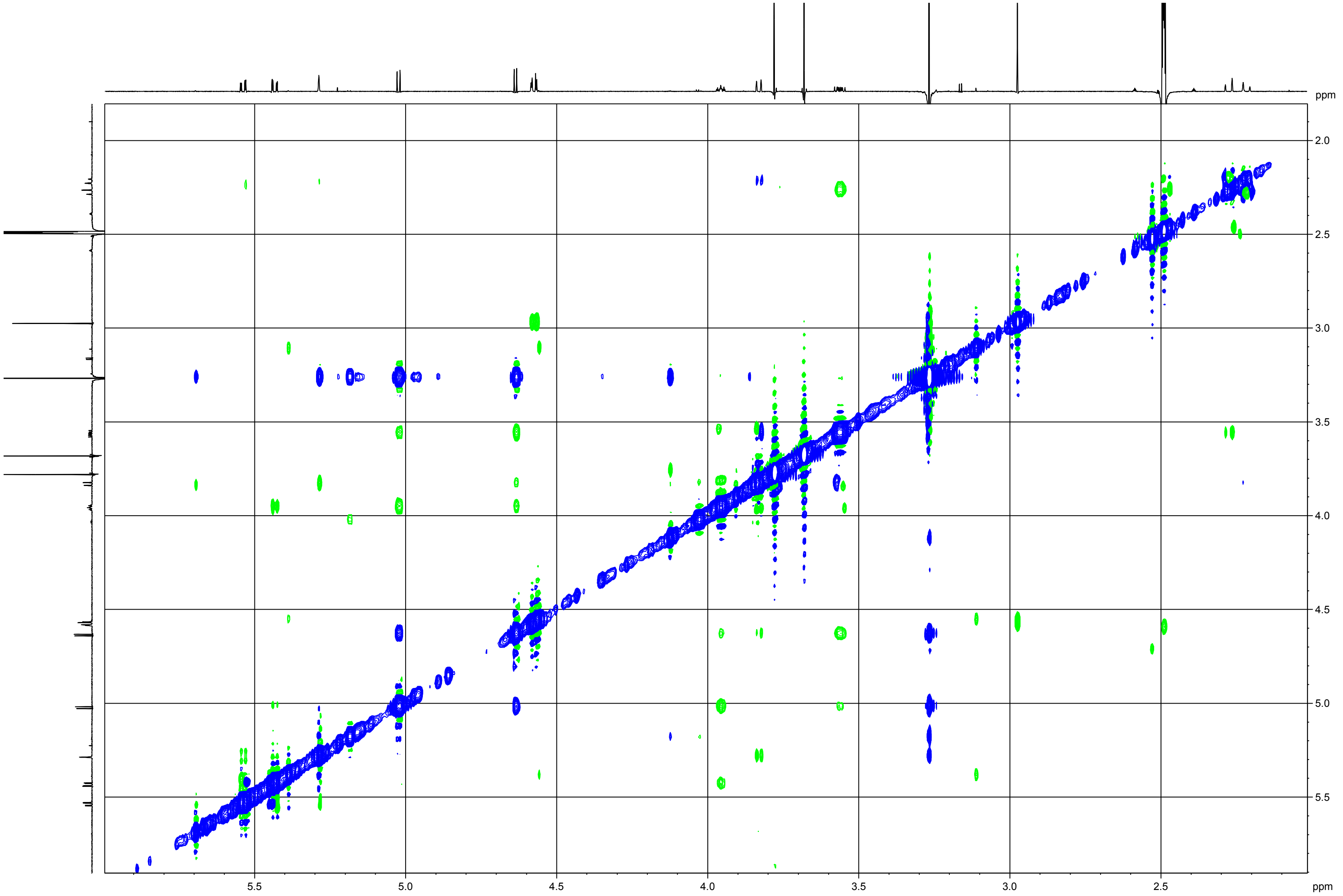

Figure S13 (continuation). ROESY (700 MHz, DMSO-d6) spectrum of pretrichodermamide E (**2**).

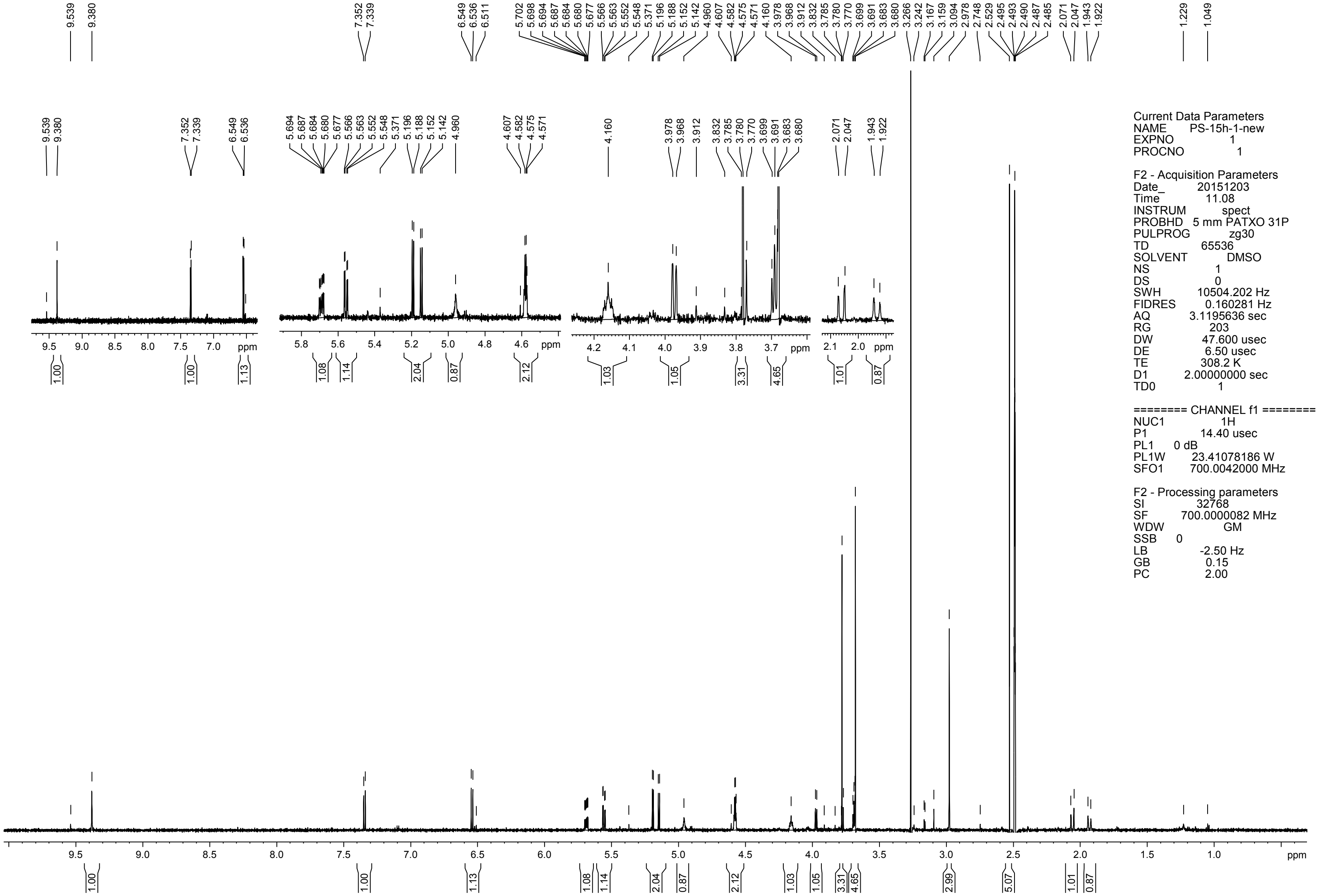

Figure S14. ^{1}H NMR (700 MHz, DMSO-d_6) spectrum of pretrichodermamide F (**3**).

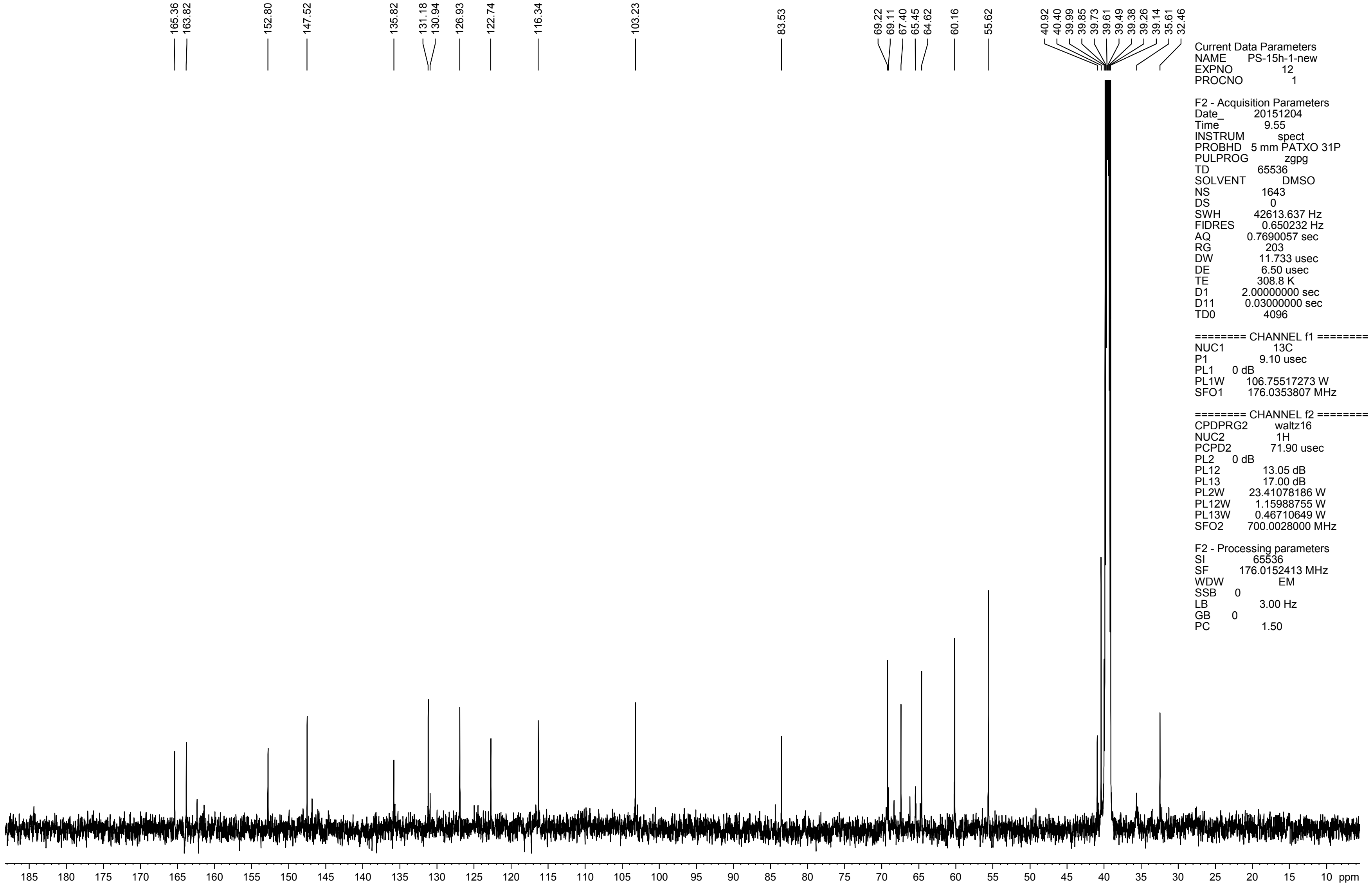

Figure S15. ^{13}C NMR (176 MHz, DMSO-d_6) spectrum of pretrichodermamide F (**3**).

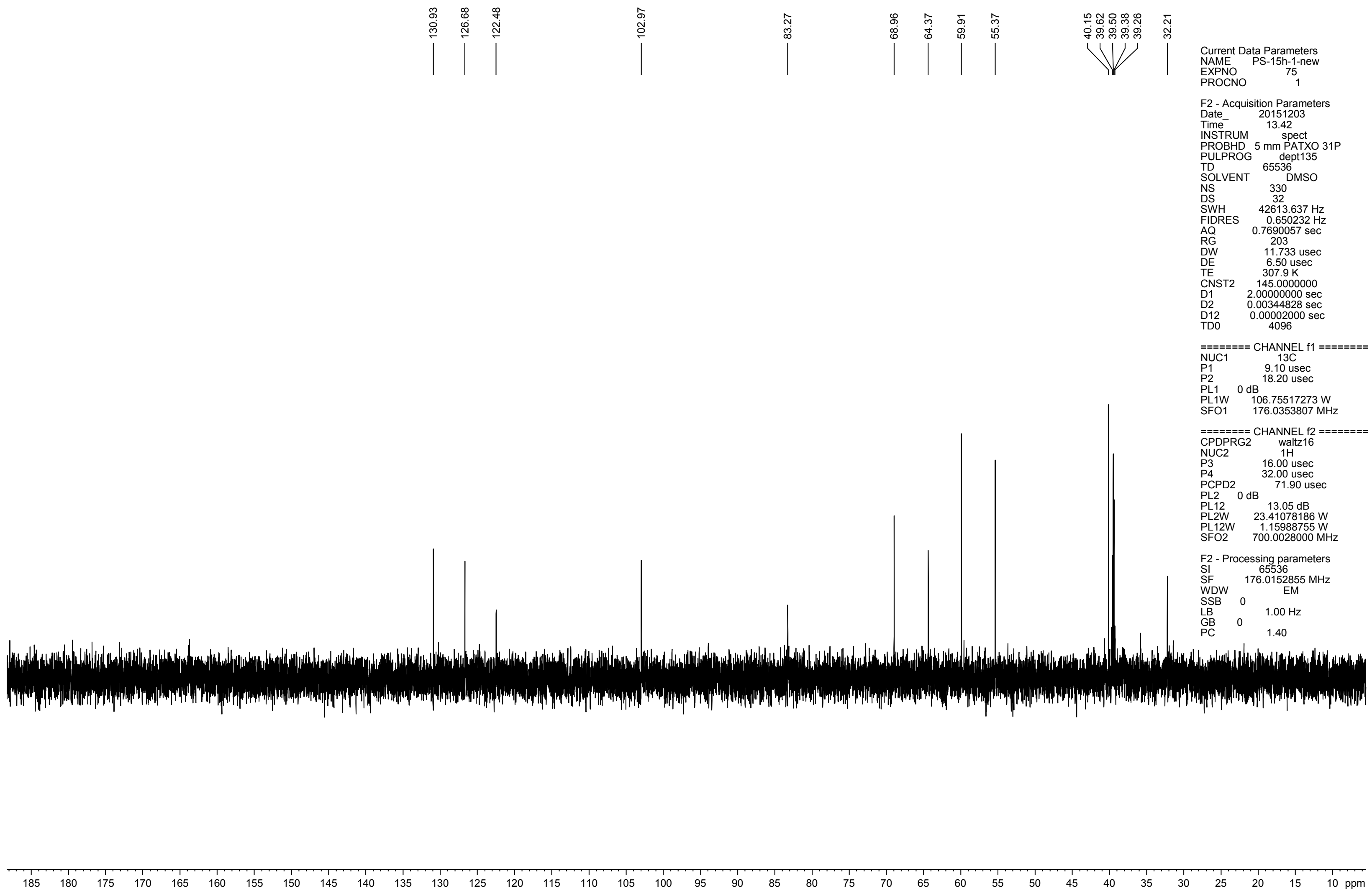

Figure S16. DEPT-135 (176 MHz, DMSO-d6) spectrum of pretrichodermamide F (**3**).

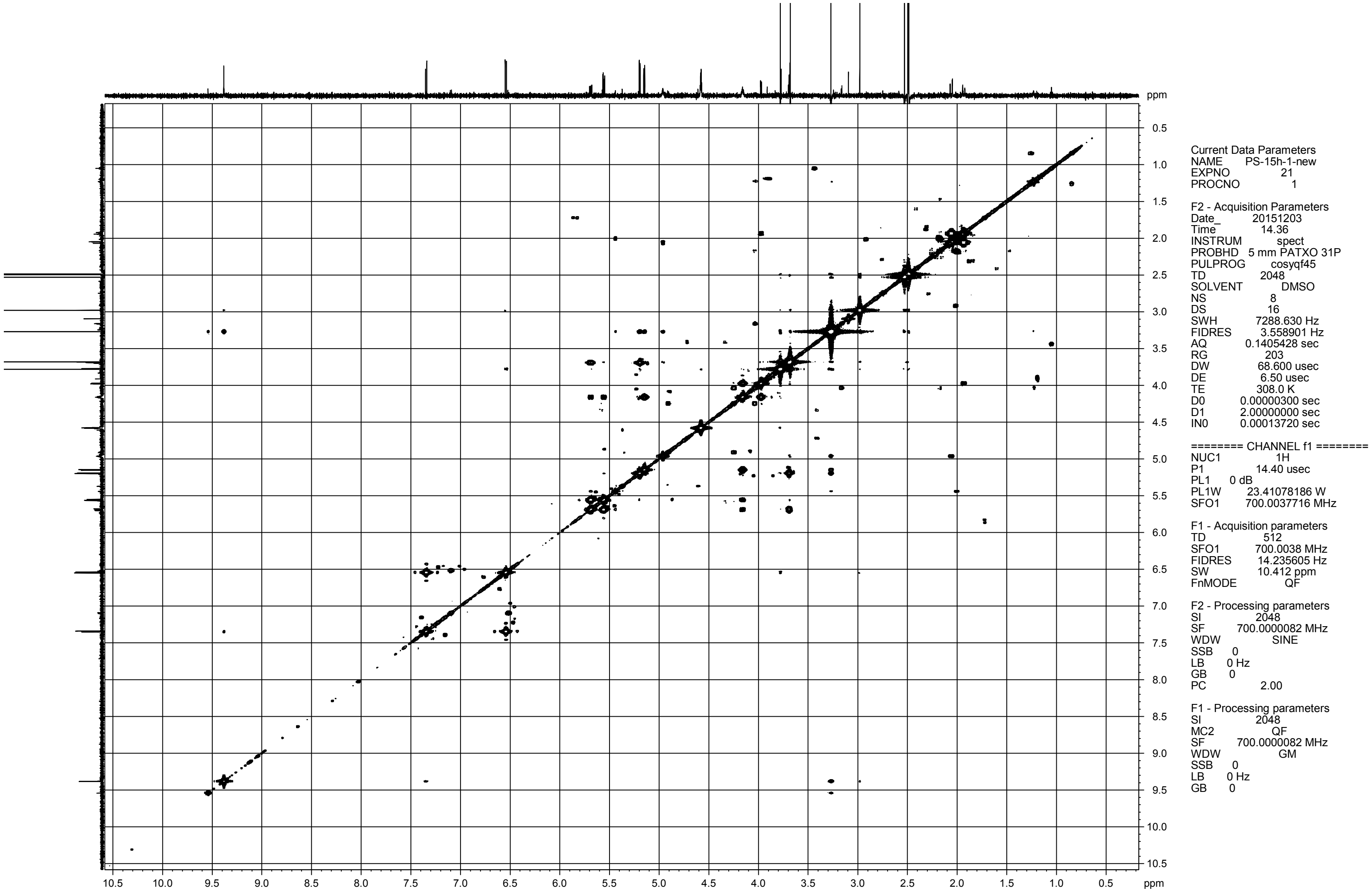

Figure S17. ^1H-^1H COSY (700 MHz, DMSO-d_6) spectrum of pretrichodermamide F (**3**).

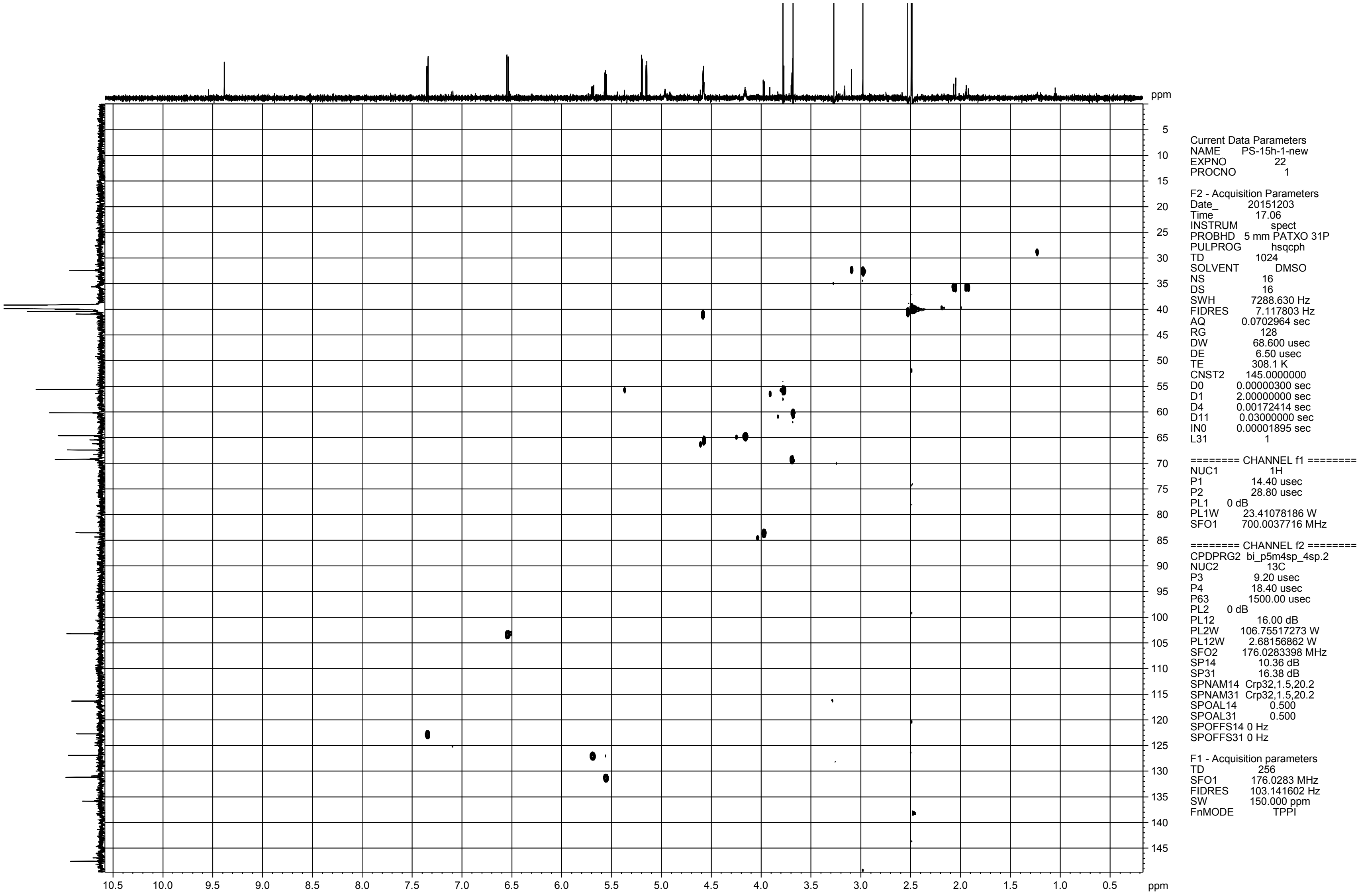

Figure S18. HSQC (700 MHz, DMSO-d6) spectrum of pretrichodermamide F (**3**).

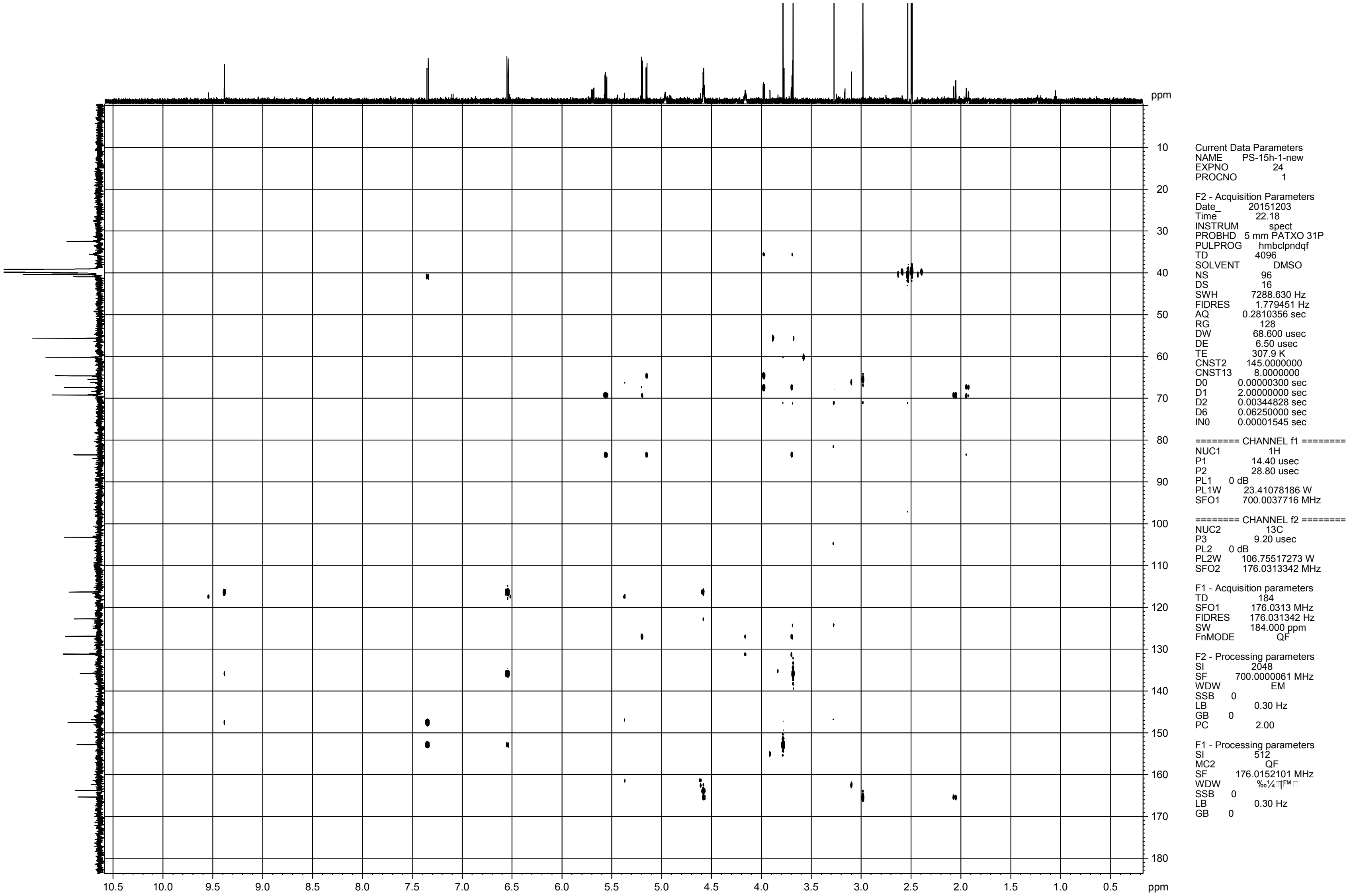

Figure S19. HMBC (500 MHz, DMSO-d6) spectrum of pretrichodermamide F (**3**).

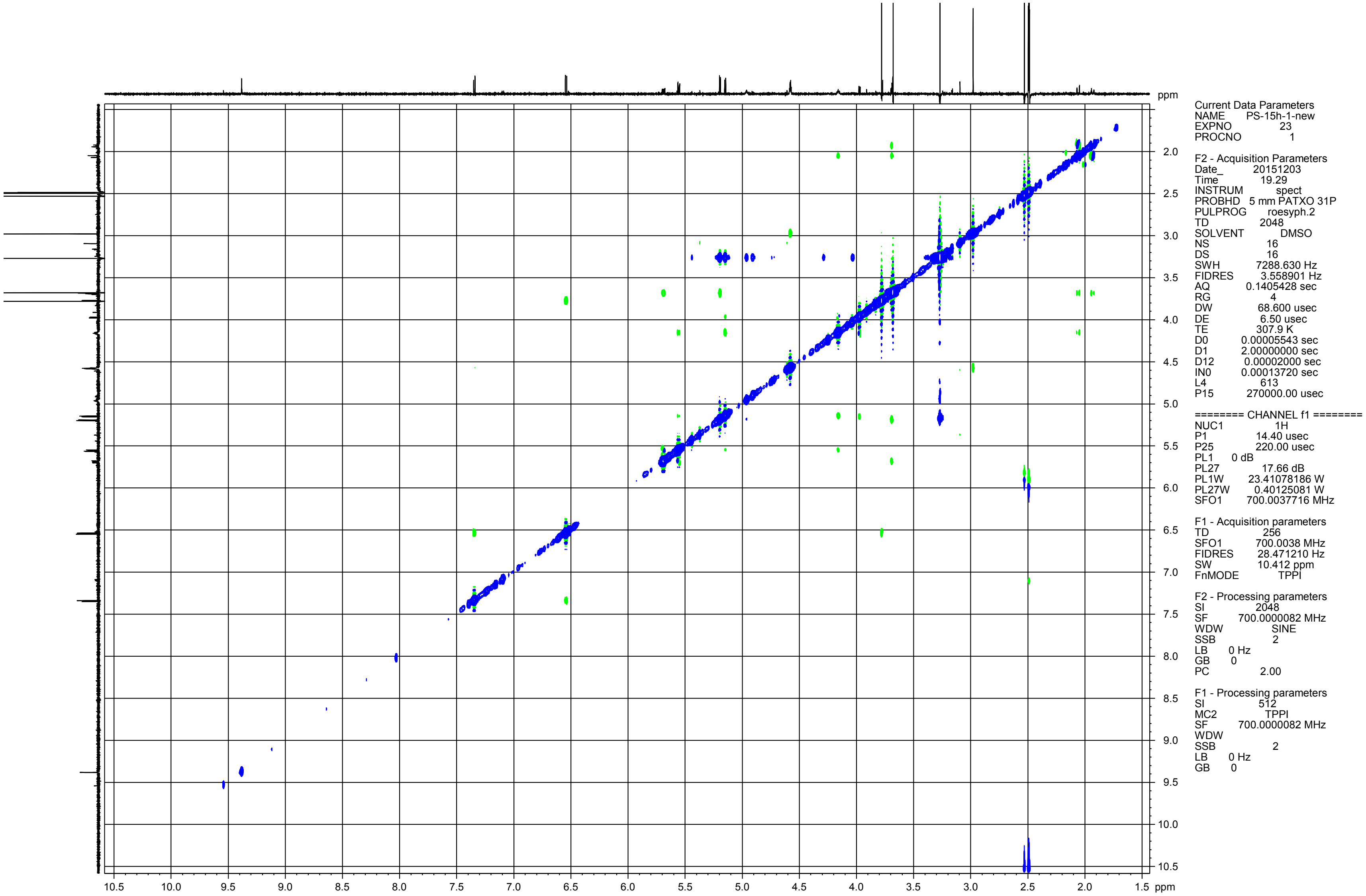

Figure S20. ROESY (700 MHz, DMSO-d$_6$) spectrum of pretrichodermamide F (**3**).

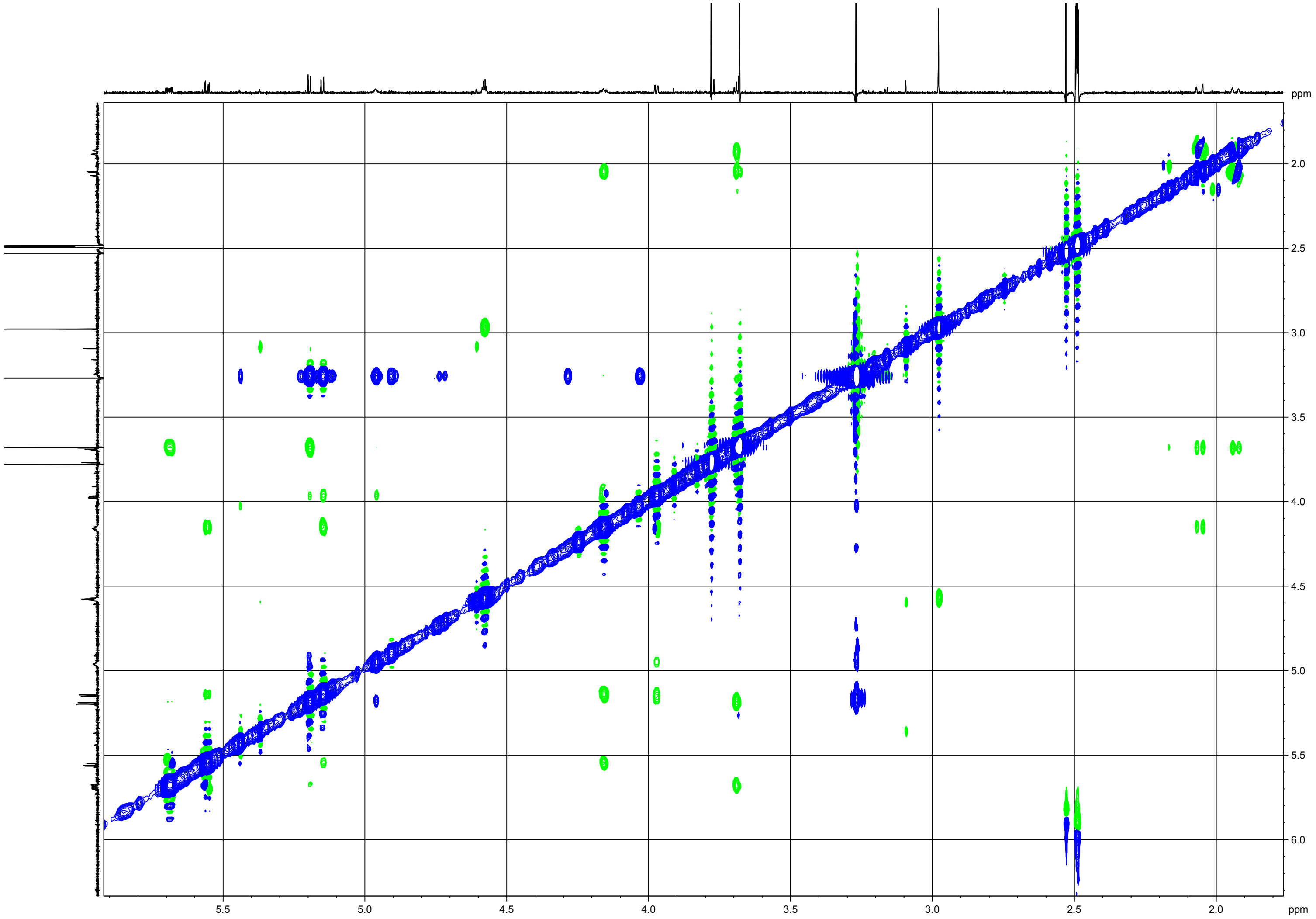

Figure S20 (continuation). ROESY (700 MHz, DMSO-d6) spectrum of pretrichodermamide F (**3**).

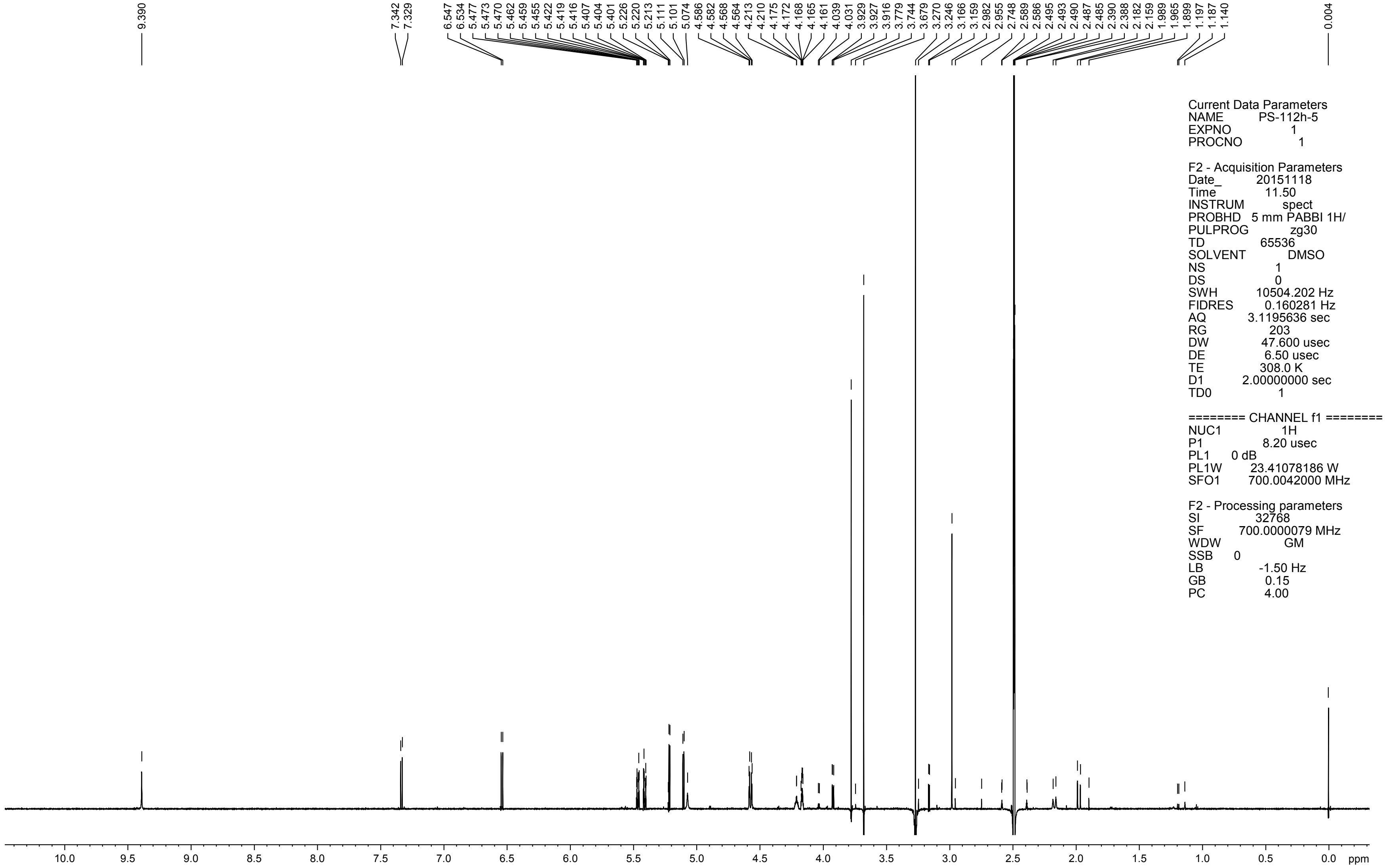

Figure S21. ^{1}H NMR (700 MHz, DMSO-d_6) spectrum of pretrichodermamide C (**4**).

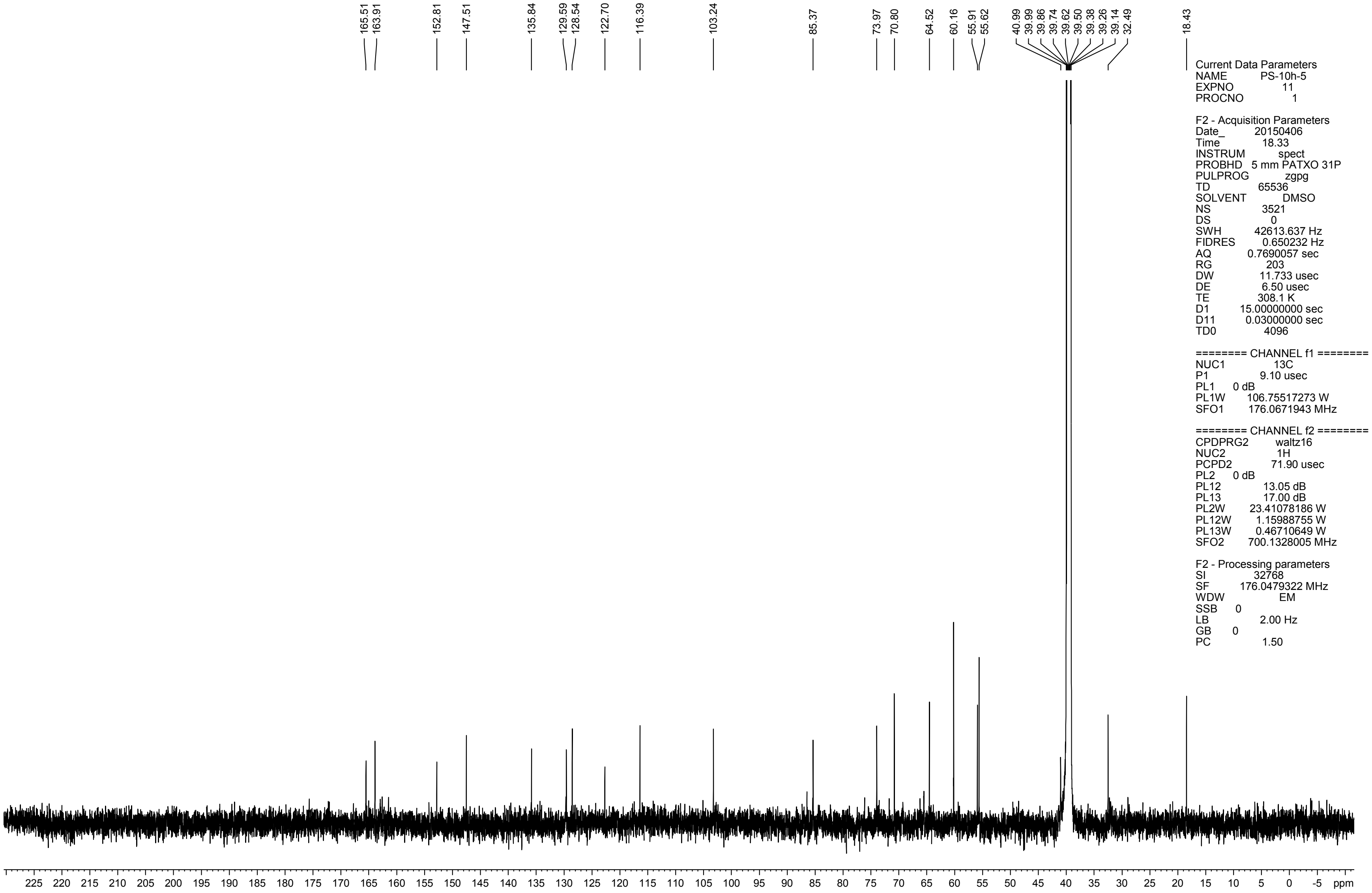

Figure S22. ^{13}C NMR (176 MHz, DMSO-d$_6$) spectrum of pretrichodermamide C (**4**).

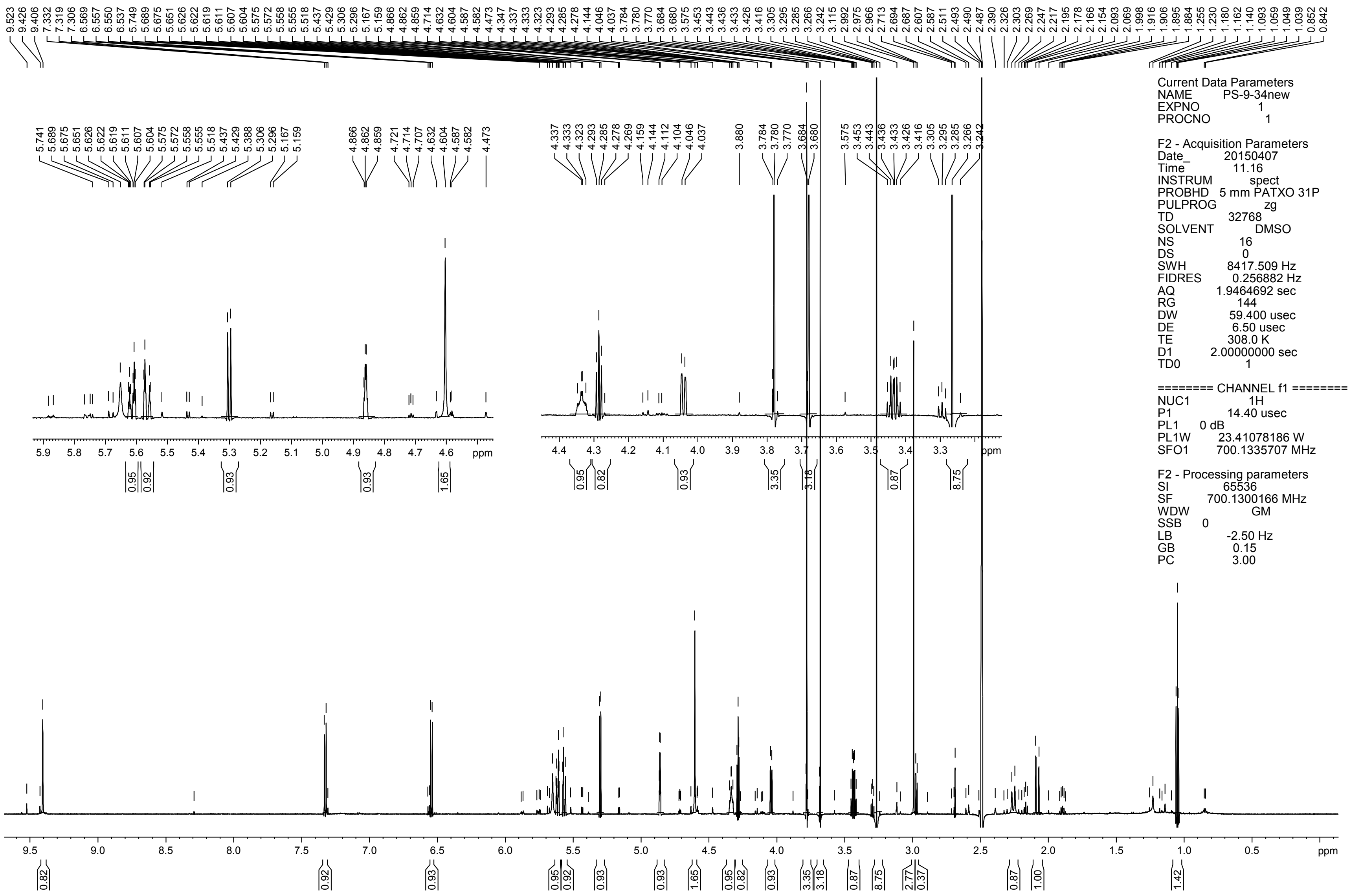

Figure S23. ^{1}H NMR (700 MHz, DMSO-d_6) spectrum of *N*-methylpretrichodermamide B (**5**)

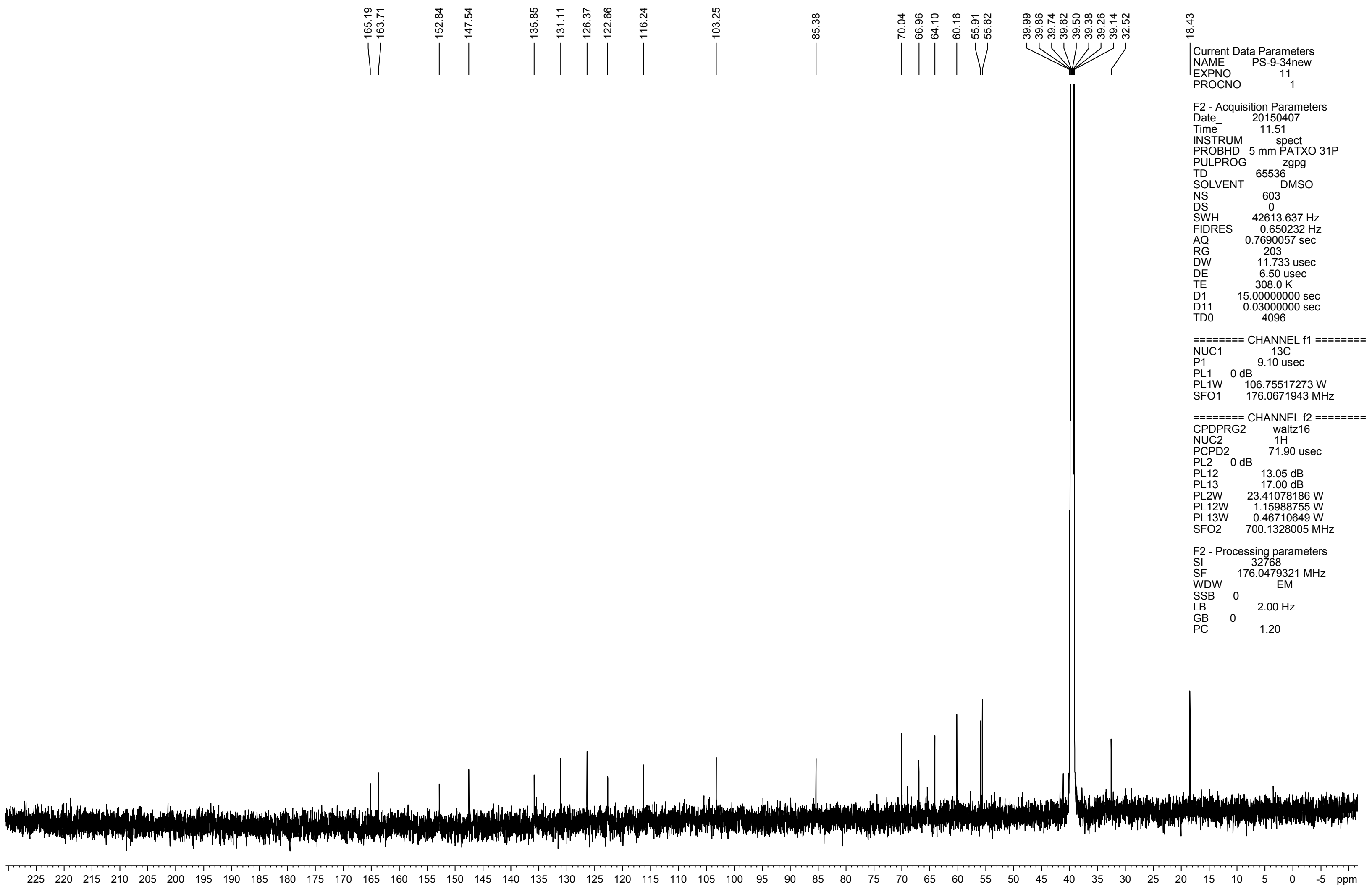

Figure S24. ^{13}C NMR (176 MHz, DMSO-d_6) spectrum of *N*-methylpretrichodermamide B (**5**).

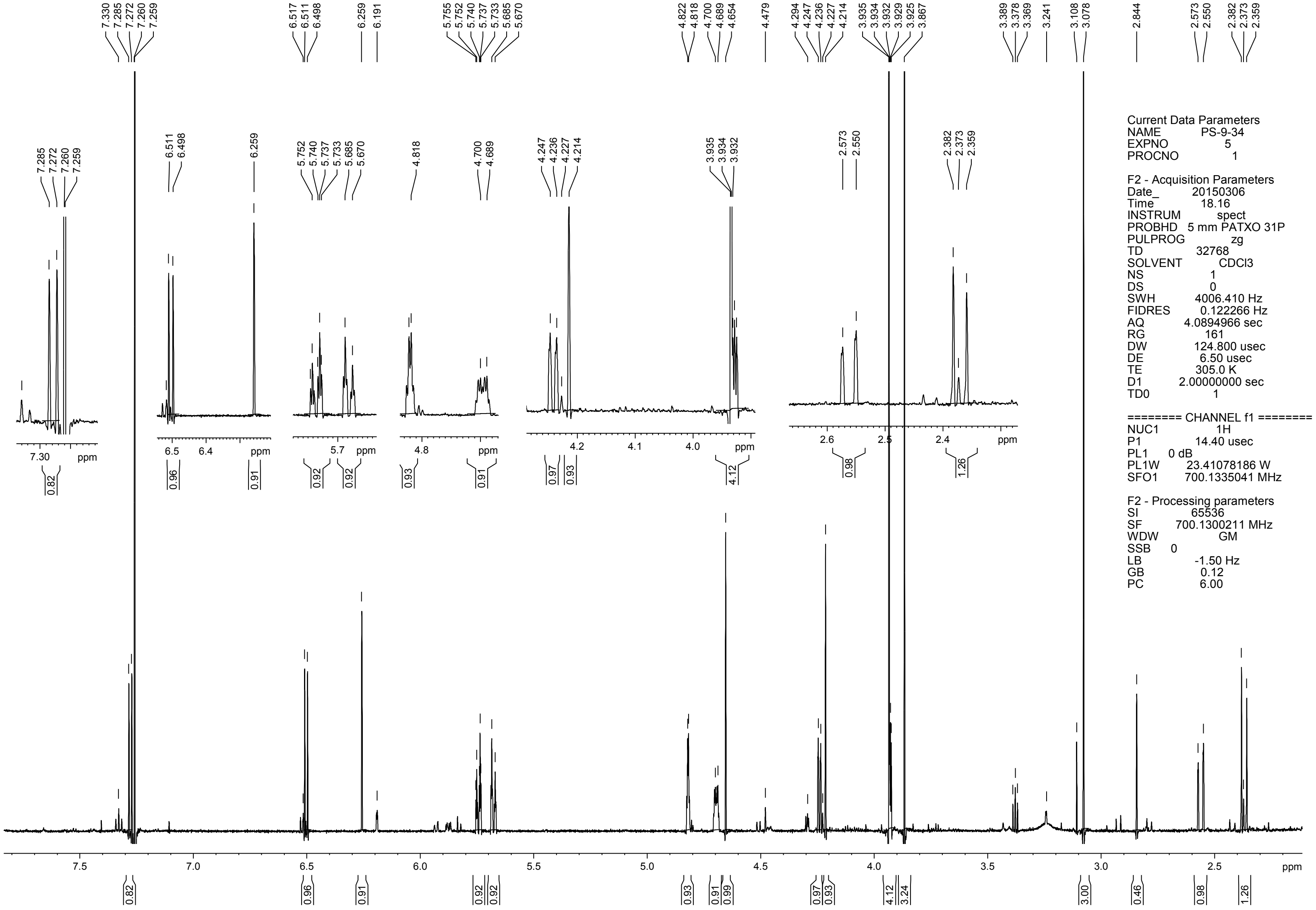

Figure S25. ^{1}H NMR (700 MHz, $CDCl_3$) spectrum of *N*-methylpretrichodermamide B (**5**).

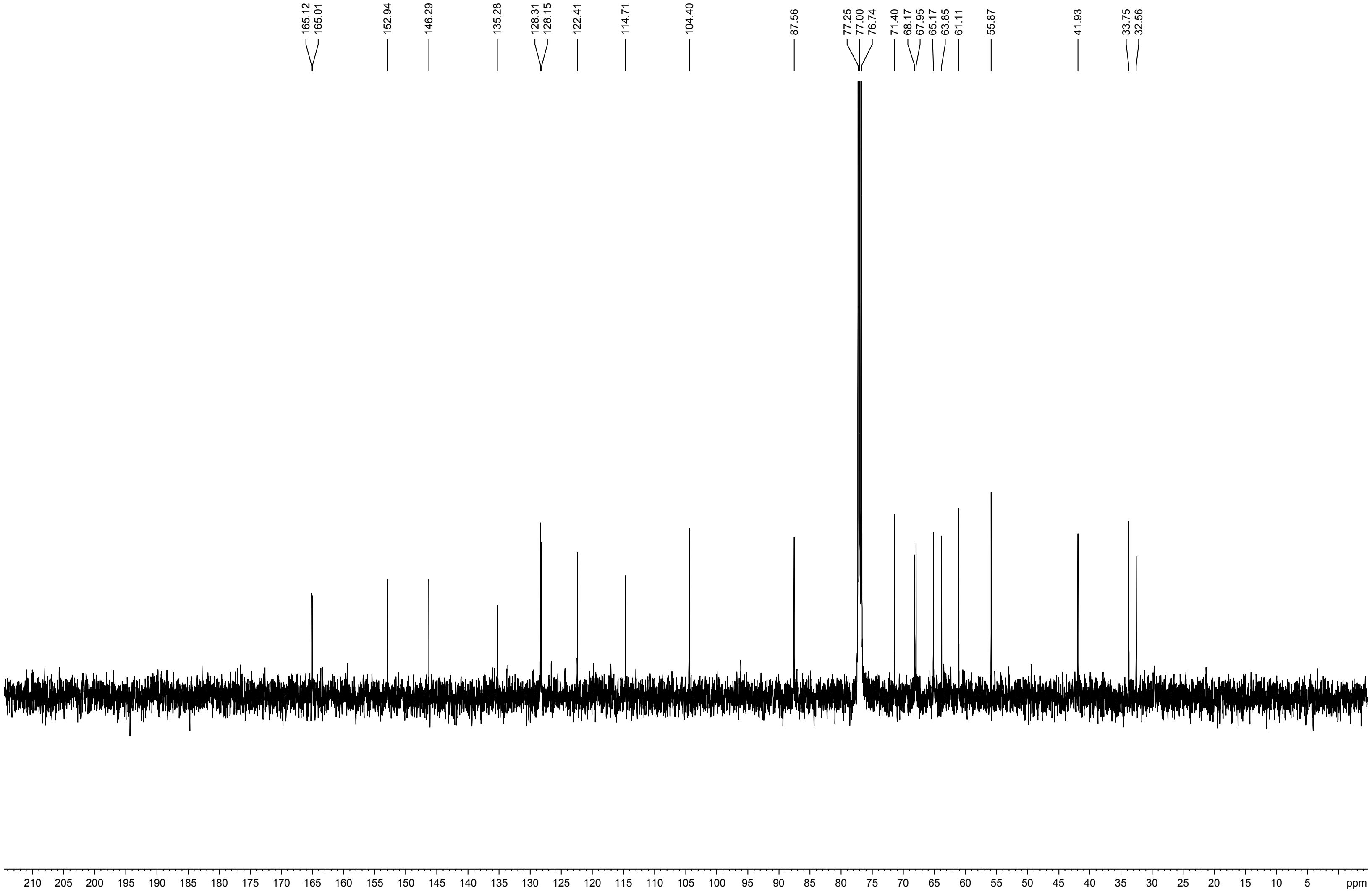

Figure S26. ^{13}C NMR (125 MHz, $CDCl_3$) spectrum of *N*-methylpretrichodermamide B (**5**).
